# Physiological tests of small airways function in diagnosing asthma: a systematic review

**DOI:** 10.1136/bmjresp-2020-000770

**Published:** 2020-12-21

**Authors:** Mohammed A Almeshari, Nowaf Y Alobaidi, Ross G Edgar, James Stockley, Elizabeth Sapey

**Affiliations:** 1 Institute of Inflammation and Ageing, University of Birmingham, Birmingham, UK; 2 Rehabilitation Health Sciences, College of Applied Medical Sciences, King Saud University, Riyadh, Saudi Arabia; 3 Respiratory Therapy Department, College of Applied Medical Sciences, King Saud Bin Abdul-Aziz University for Health Sciences, Al Ahsa, Saudi Arabia; 4 University Hospitals Birmingham NHS Foundation Trust, Birmingham, UK

**Keywords:** asthma, lung physiology

## Abstract

**Background:**

Asthma is a common, heterogeneous disease that is characterised by chronic airway inflammation and variable expiratory airflow limitation. Current guidelines use spirometric measures for asthma assessment. This systematic review aimed to assess whether the most commonly reported tests of small airways function could contribute to the diagnosis of asthma.

**Methods:**

Standard systematic review methodology was used, and a range of electronic databases was searched (Embase, MEDLINE, CINAHL, CENTRAL, Web of Science, DARE). Studies that included physiological tests of small airways function to diagnose asthma in adults were included, with no restrictions on language or date. The risk of bias and quality assessment tools used were Agency for Healthcare Research and Quality tool for cross-sectional studies and Quality Assessment of Diagnostic Accuracy Studies 2 for diagnostic test accuracy (DTA) studies.

**Results:**

7072 studies were identified and 10 studies met review criteria. 7 included oscillation techniques and 5 included maximal mid-expiratory flow (MMEF). Studies were small and of variable quality. In oscillometry, total resistance (R5) and reactance at 5 Hz (X5) was altered in asthma compared with healthy controls. The percentage predicted of MMEF was lower in patients with asthma compared with controls in all studies and lower than the % predicted forced expiratory volume in 1 s. In DTA of oscillometry, R5 showed a sensitivity between 69% and 72% and specificity between 61% and 86%.

**Conclusion:**

There were differences in the results of physiological tests of small airway function in patients with asthma compared with controls. However, studies are small and heterogeneous. Further studies are needed to assess the effectiveness of these tests on a larger scale, including studies to determine which test methodology is the most useful in asthma.

**Key messages**

- Is there evidence to support the use of physiological tests of small airways function in the diagnosis of asthma?
- There is evidence of small airways dysfunction in asthma, which some physiological tests can identify. However, studies are small and heterogeneous and more studies are needed to understand the clinical utility of these tests.
- This systematic review provides a summary of the current evidence around physiological tests of small airways and asthma. It includes recommendations for the future work required to before the adoption of physiological small airways tests in the diagnosis of asthma.

## Background

Asthma is a common but heterogeneous disease characterised by chronic airway inflammation and clinically defined by the presence of respiratory symptoms that vary over time and in intensity. Physiologically, asthma is characterised by variable expiratory airflow limitation which may become persistent over time.^1^ Symptoms and airflow limitation can be extremely variable, including the age of onset, triggers for symptoms, the decline in lung function and therapeutic response.

It is estimated that 339 million people are affected by asthma globally^1^ but diagnosing asthma is often challenging as there is no gold standard test. This has led to a high burden of undiagnosed disease, especially in children and older adults.^2 3^ According to current guidelines,^1 4^ a diagnosis of asthma should be objectively supported with an assessment of forced expiratory volume in 1 s (FEV_1_) reversibility. However, some patients with asthma have no evidence of reversibility or airflow obstruction^5 6^ and airflow obstruction and reversibility are seen in patients with alternative diagnoses such as chronic obstructive pulmonary disease (COPD).^7 8^ Furthermore, the forced manoeuvres required for spirometry requires effort and coordination, which can be difficult for some individuals.^9^

In the past, asthma was thought to only affect larger airways^10^ but current evidence suggests that small airways (defined as airways of ≤2 mm in diameter) are affected as well. The small airways may form a site of active disease, even in the absence of airflow obstruction.^11^ If the small airways are the first to be affected in asthma, identifying small airways dysfunction (SAD) may help identify asthma earlier, enabling treatment. However, there are a large number of tests that report small airways function. Some of these are being used as secondary outcomes in experimental studies of asthma, to determine asthma phenotype and assess the response to new therapies.^12^ The evidence to support the use of any physiological test of small airways function in the diagnosis of asthma is unclear.

This systematic review aimed to assess the evidence to support the use of commonly reported physiological tests of small airways function to diagnose asthma in adults, and assess if the selected tests should be included in future clinical studies of the disease.

## Methods

The protocol was prospectively registered in the international registry of systematic reviews (PROSPERO) with registration number CRD42019133239. The review was prepared in accordance to the Preferred Reporting Items for Systematic Reviews and Meta-Analyses (PRISMA) guidelines^13^ and the PRISMA checklist is provided in online supplemental material file S1. Meta-analysis was considered where homogenous results were provided, otherwise data were pooled for graphical presentational purposes.

10.1136/bmjresp-2020-000770.supp1 Supplementary data

Through both scoping searches and discussion with experts, the following test were selected to be included in the search, forced oscillation technique (FOT), impulse oscillometry (IOS) and maximal mid-expiratory flow (MMEF) also known as forced expiratory flow between 25% and 75% of forced vital capacity (FVC) (FEF_25%–75%_), and multiple breath washout test (MBW). These tests were selected as they represented some of the most commonly reported physiological tests of small airways function in obstructive lung disease in adults. Online supplemental figure S2 shows the Population, Intervention, Comparison and Outcome (PICO) chart with the studies selection criteria.

10.1136/bmjresp-2020-000770.supp2 Supplementary data

### Eligibility criteria

Studies were considered for inclusion if they used one of the proposed physiological small airways function tests (FOT/IOS, MBW, MMEF) in diagnosing asthma in adults aged >18 years old. Patients with either a physician diagnosis or a suspected diagnosis of asthma were considered for inclusion. FEV_1_ was used as the comparator as it is the current standard in physiological airway assessment. Studies were excluded if they included only children (<18 years), patients with respiratory infections within 2 months of the assessment, did not assess FEV_1_, included patients with asthma-COPD overlap, were laboratory-based studies, animal-based studies or case series of less than 10 participants. There were no language or publication date restrictions.

Search queries were carried out in May 2019 (and the detailed search strategy is found in online supplemental material file S3) on the following electronic databases: Embase, MEDLINE, CINAHL, Cochrane Central Register of Controlled Trials (CENTRAL), Web of Science (Abstracts and Proceedings) up to 5 years and DARE database for grey literature. Clinicaltrials.gov and EudraCT were also searched for active trials or published data. Hand searching of references listed in the selected articles was included. Search terms contained subject heading and terms for the selected test (IOS/FOT, MBW and MMEF) combined with terms of asthma and small airways function.

10.1136/bmjresp-2020-000770.supp3 Supplementary data

### Study selection

Search results were imported into EndNote 9.1 (Clarivate Analytics) where duplicates were removed and data was uploaded to Rayyan^14^ (a webapp tool used for screening titles and abstracts). Abstracts were screened blindly and independently by the authors MA and NYA using the predefined inclusion and exclusion criteria. Disagreements were resolved by discussion, otherwise by the third reviewer, whose initials were RGE. Full-text articles were acquired and imported into EndNote 9.1 by author MA and similar abstract screening methodology was used in screening full texts for eligibility.

### Data extraction

Data were extracted by author MA and checked by author NYA for consistency and accuracy using a custom, piloted data extraction form. Diagnostic criteria used to identify asthma, tests used to aid the diagnosis such as airway reversibility, asthma severity, phenotype, medications, the device used and comorbidities were extracted to aid narrative review and provide clinical context. Studies were categorised based on the small airways test used. In diagnostic test accuracy (DTA) studies, sensitivity and specificity values were extracted and a 2×2 contingency table was calculated.

#### Quality and risk of bias assessment

Quality and risk of bias were assessed using validated tools based on study design. Cross-sectional studies were assessed using the Agency for Healthcare Research and Quality (AHRQ) checklist tool.^15^ The Quality Assessment of Diagnostic Accuracy Studies 2^16^ (QUADAS-2) was used in DTA. The QUADAS-2 tool assesses the risk of bias of studies over four domains: flow and timing, reference standard, index test and patient selection. The tool also assesses for applicability concerns under three domains: reference standard, index test and patient selection.

#### Descriptions of the tests of small airways function included in the reported studies

Here, only tests included in the analysed studies are described.

### Oscillometry

Oscillometry can be assessed using either the FOT or IOS. Oscillometry transmits oscillating sound signals of various frequencies along the bronchial tree, providing a measure of the total airway resistance (resistance at 5 Hz (R5)) and the proximal airway resistance (resistance at 20 Hz (R20)), which allows for the derivation of small airways resistance (R5–R20). Reactance at 5 Hz (X5) relates to physical properties of the lung parenchyma and its ability to expand and facilitate alveolar filling. Resonant frequency (Fres) is the point at which reactance is zero (when forces of inertia and capacitance are equal). The area of reactance (AX) is the sum of area under the reactance curve between X5 and Fres.^17 18^ Limitations with this technique include the lack of universal normal ranges for all populations and variance of results between different devices, which can impede interpretation.^19^

### Maximum mid-expiratory flow

The MMEF is the mean forced expiratory flow between 25% and 75% of the FVC (FEF_25%–75%_) and is taken from the spirometric blow with the largest sum of FEV1 and FVC. The MMEF is highly dependent on the validity of the FVC measurement and the level of expiratory effort.^20 21^ MMEF is commonly reported in studies of small airways as it is readily accessible from spirometry reports.

### Patient and public involvement

Due to the nature of the study design, patients and public were not involved in this systematic review.

## Results

### Study selection

**Figure 1:**
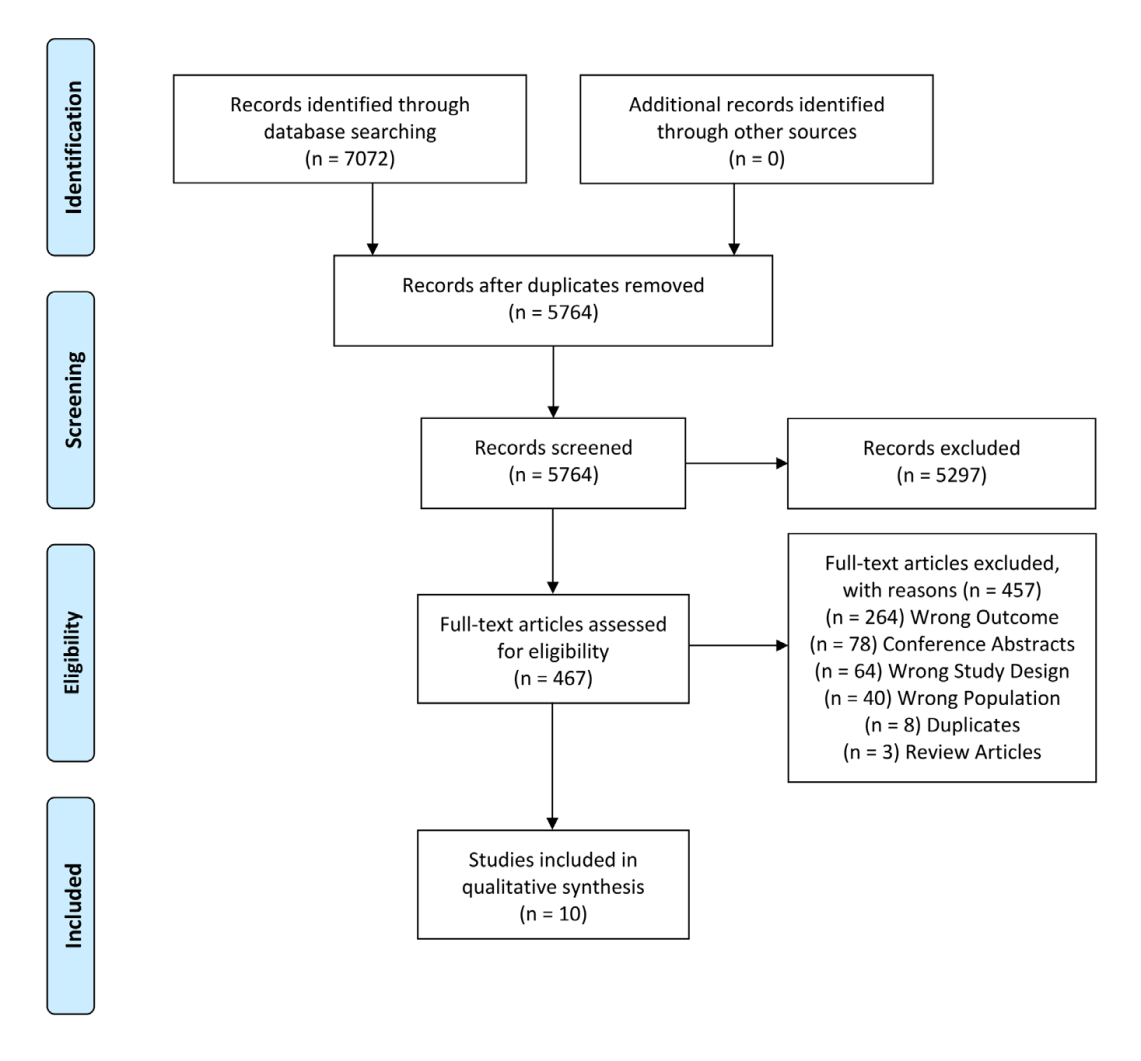
Preferred Reporting Items for Systematic Reviews and Meta-Analyses flow chart of included studies.

Initial searches identified 7072 abstracts. After the removal of duplicates, 5764 abstracts were screened of which 469 abstracts included for full text screening. Ten articles ultimately met the inclusion criteria (figure 1 shows the Preferred Reporting Items for Systematic Reviews and Meta-Analyses flow diagram). Articles excluded in the full-text screening phase are described in online supplemental material file S4 with reasons given. All included studies were cross-sectional in design and 3/10 of the included studies were DTA studies.

10.1136/bmjresp-2020-000770.supp4 Supplementary data

### Study characteristics

Seven of the included articles reported oscillometry (IOS/FOT)^22–28^ and five reported MMEF.^23 29–31^ None of the included studies reported MBW use in diagnosing asthma. Seven studies were not designed as DTA studies and are presented in table 1. Three studies were designed as DTA studies and these are presented in table 2. The diagnostic criteria used to confirm a diagnosis of asthma differed among studies. Four studies used Global Initiative for Asthma guidelines,^24 25 28 30^ one American Thoracic Society guidelines^27^ and one the global strategy: Joint Report of the National Institute for Heart, Lungs and Blood and WHO.^29^ Three studies recruited patients based on a previous diagnosis of asthma, without reporting the diagnosis criteria used.^22 23 26^ One study reported that patients with symptoms of asthma were included without any formal diagnosis.^31^ All included studies were based in different countries (the USA, UK, Japan, Korea, Turkey, Egypt, Russia, Serbia, Iran and China) from four different continents (North America, Europe, Asia and Africa) making the ethnicity of participants heterogeneous. Body mass index (BMI) was only reported in three of the included studies.^23–25^ Meta-analysis of the data were inappropriate due to the variety and scope of methodological design. Where appropriate, data were displayed graphically to aid the representation of results. No MMEF studies explicitly corrected for FVC, which can potentially affect interpretation.

**Table 1:** The characteristics of included studies without a diagnostic test accuracy analysis

ID	Sample size	Age	BMI	SA device	SA function	FEV_1_	Diagnosis criteria
Mendonça *et al*^23^ USA Cross-sectional	Asthma 35 Control 34	21 (3) 22 (2)	66 kg (12)*	NR	**Asthma**: MMEF% 69 (20) R8 cmH_2_O 2.91 (0.99) **Control**: MMEF 93% (20) R8 cmH_2_O 2.21 (0.48)	88 (11) 95 (10)	Previous diagnosis of asthma
Mori *et al*^24^ Japan Cross-sectional	Asthma 49 Control 13	53 (15) 42 (16)	23.9 (6.3) 21.2 (3.3)	FOT- MostGraph-01	**Asthma**: MMEF% 49.9 (27.7) X5 cmH_2_O −0.96 (1.03) R5–R20 cmH_2_O 0.86 (0.62) R5 cmH_2_O 4.2 (1.43) **Control**: MMEF 88.9 (22.1) X5 cmH_2_O −0.05 (0.27) R5–R20 cmH_2_O 0.32 (0.44) R5 cmH_2_O 3.29 (1.17)	80.5 (20.2) 105.4 (17.8)	GINA 2009
Mousa and Kamal^25^ Egypt Cross-sectional	Asthma 25 Control 20	45 (13) 34 (13)	29.27 (6.03) 26.6 (4.97)	IOS- Masterscreen	**Asthma**: R5% 245.24 (109.18) X5 (Unit NR) −2.87 (1.84) **Control**: R5% 109.25 (19.40) X5 (Unit NR) −0.28 (0.10)	59.68 (23.73) 89.75 (8.70	GINA 2017
Koruga *et al*^22^ Serbia Cross-sectional	Asthma 31	23 (5)	NR	IOS- Masterscreen	**Asthma**: X5 kPa −0.09 (0.05) AX kPa 0.23 (0.16) R5 kPa 0.34 (0.09)	4.47 L (0.64)	Previous diagnosis of asthma
Nair *et al*^26^ UK Cross-sectional	Asthma 82 Control 61	49 (17) 28 (10)	NR	IOS- Masterscreen	**Asthma**: X5% 441.72 (137.86) R5% 162.22 (7.5) **Control**: X5% −229.8 (125.75) R5% 111.01 (3.96)	83.99 (2.23) 99.25 (1.72)	Previous diagnosis of asthma
Gulden *et al*^30^ Turkey Cross-sectional	Asthma 443	37 (15)	NR	Vmax 229	MMEF L/s 3.17 (5.8)	2.99 (0.9)	GINA 2006
Son *et al*†^31^ Korea Cross-sectional	Asthma 125	43 (1)	NR	NR	1. MMEF% 97.67 (3.48) 2. MMEF% 95.08 (5.74) 3. MMEF% 70.16 (4.64)	1. 107.84 (1.79) 2. 105.2 (3.43) 3. 96.16 (2.71)	Clinical suspicion of asthma

Values reported in mean (SD).

*Weight in kg.

†Values reported in mean (SEM).

BMI, body mass index; FEV_1_, forced expiratory volume in 1 s; FOT, forced oscillation technique; GINA, Global Initiative for Asthma; ID, study identification (authors) country, research type; IOS, impulse oscillometry; NR, not reported; SA, small airways.

**Table 2:** The characteristics of included studies with a diagnostic test accuracy analysis

ID	Sample size	Age	SA device	FEV_1_	SA test	Cut-off	TP	FN	FP	TN	Sen. %	Spc. %	Diagnosis criteria
Li *et al*^27^ China Cross-sectional	Asthma 561 Control 205	50.5 (18.8)*	MasterLab-IOS	NR	R5 X35	NR	404 297	157 264	80 49	125 156	72 53	61 76	ATS guidelines
Nikkhah *et al*^28^ Iran Cross-sectional	Asthma 87 Control 87	41.4 (15.5) 37.6 (17.8)	MasterScreen-IOS	2.2 (0.6) L 3.2 (0.9) L	R5 X5	>0.51 ≤−0.2	60 36	27 51	12 8	75 79	69 41	86 91	GINA 2008
Iartsev^29^ Russia Cross-sectional	1. Asthma 209 2. Asthma 75 3. Asthma 81 Control 216	1. 47.4 (0.8) 2. 47.4 (0.9) 3. (1.6) 43.8 (0.8)	Jaeger Master Screen	1. 101 (0.9) 2. 70 (0.73) 3. 53.1 (1.6) 102.6 (1.4)	MMEF all groups	90% 70% 50%	138 74 80	71 1 1	19 0 0	197 216 216	66 99 99	91 100 100	Global strategy: Joint Report of the NHLBI and WHO 1993

*For both groups.

ATS, American Thoracic Society; FEV_1_, forced expiratory volume in 1 s; FN, false negative; FP, false positive; GINA, Global Initiative for Asthma; ID, study identification (authors) country, research type; NHLBI, National Heart, Lung, and Blood Institute; NR, not reported; SA, small airways; Sen, sensitivity; Spc, specificity; TN, true negative; TP, true positive.

### Risk of bias

**Figure 2:**
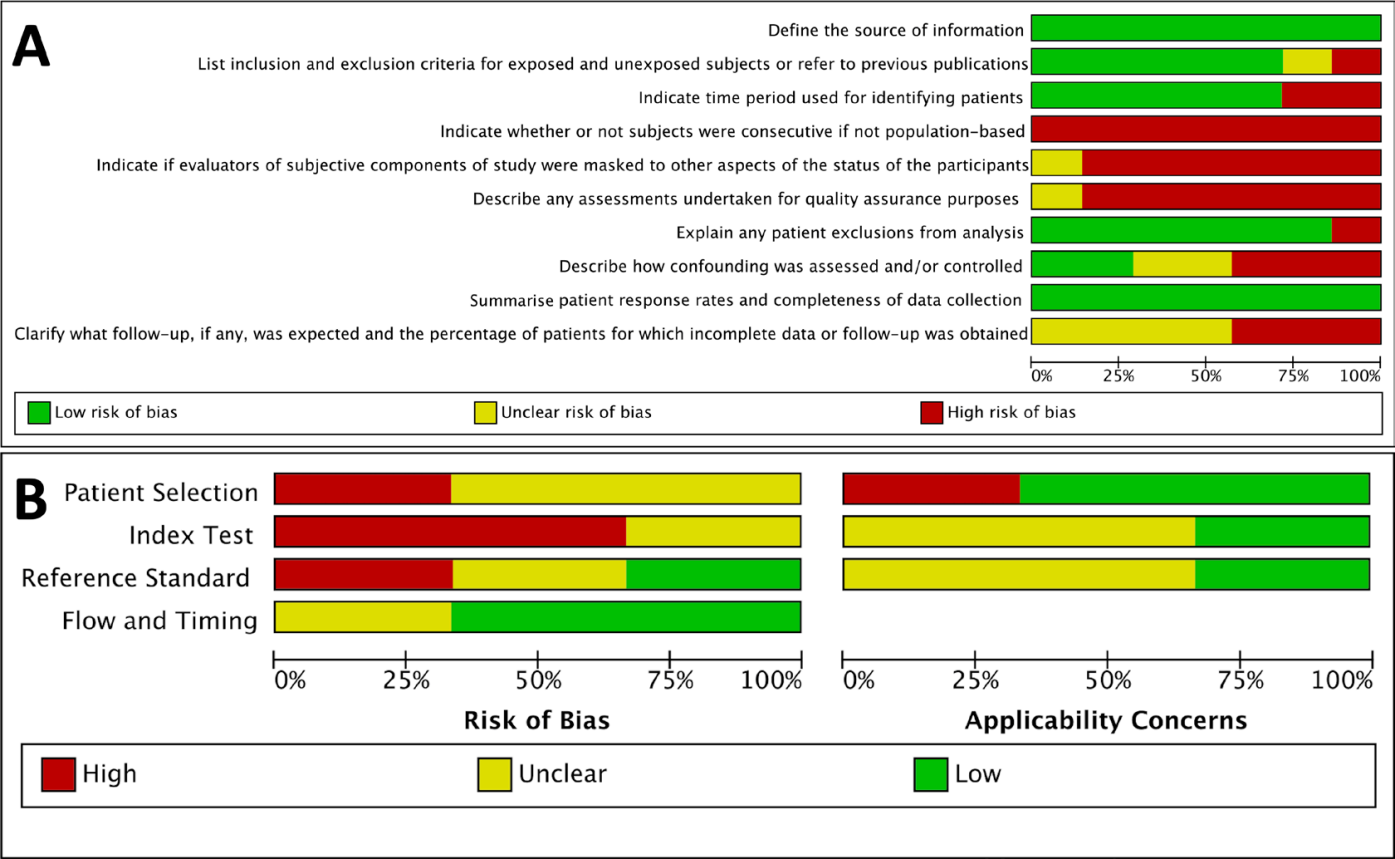
(A) Graph of Agency for Healthcare Research and Quality tool for quality/risk of bias of cross-sectional studies. (B) Graph of Quality Assessment of Diagnostic Accuracy Studies 2 tool for diagnostic test accuracy studies.

Two risk of bias and quality assessment tools were used in this systematic review, based on the design of the included studies. Seven studies were assessed using the AHRQ tool for cross-sectional studies^15^ (see figure 2A). This highlighted potential methodological issues around subject selection and quality assurance concerns, which may have impacted on the reliability of results and the reporting of study follow-up. There was an overall low risk of bias around patient recruitment (including the source of subjects), the inclusion/exclusion criteria and time periods when patients were identified. Response rates and completeness of results were all reported. A summary of all included studies using both tools is available in online supplemental material file S5.

10.1136/bmjresp-2020-000770.supp5 Supplementary data

Three studies were assessed using QUADAS-2 tool for DTA studies (see figure 2B). One study had a high risk of bias and applicability concern in the patient selection phase. Two studies had a high risk of bias in the index test.

### Results of individual studies

#### Oscillometry

Seven studies used oscillometry. One study used FOT,^24^ five studies used IOS^22 25–28^ and one study did not reported which type of oscillometry was used.^23^ Five studies reported R5,^22–26^ which represent the total lung resistance. Only one study reported R5–R20.^24^ The values of the test were reported in different units with Mori *et al*^24^ and Mendonça *et al*^23^ reporting results in cmH_2_O/L/s, Nair *et al*^26^ and Mousa and Kamal the % predicted values and Koruga *et al*^22^ and Nikkhah *et al*^28^ in kPa/L/s.

Mori *et al*^24^ reported R5, R5–R20, X5 and MMEF in 49 asthmatic patients,13 controls and 51 COPD patients. They described differences in MMEF, R5-R20 and X5 when comparing asthma to control subjects but not R5. In addition, they reported that the coloured three-dimensional model provided by the FOT device could differentiate between asthma, COPD and healthy subjects, with a higher resistance and lower reactance observed in asthma. Asthma severity was not reported and 24 of the asthmatic subjects were ex-smokers.

Mendonça *et al*^23^ studied 35 asthmatic and 34 non-asthmatic participants but used a different technique and frequency than the commonly reported value. Oscillometry values were reported in cmH_2_O/L/s. The whole breath resistance at 8 Hz (R8) and the minimum resistance at maximum inhalation R_min_ were both different when comparing the asthmatic (R8=2.91±0.99) and non-asthmatic group (R8=2.21±0.48). In the asthmatic patients, both the MMEF % predicted value was lower (69%±20) than healthy controls (93%±20) and a higher R_min_ was observed. They also conducted a methacholine challenge test (MCT) in all participants and found that (31/35) asthmatic subject and (8/34) non-asthmatic group had a positive result. A subgroup analysis was reported including asthmatics with positive MCT (31/35) and non-asthmatic with a negative MCT (26/34) and similar results were reported to the overall analysis with a higher R_min_ in positive MCT (1.41±0.42) compared with (1.02±0.24) in negative MCT. Moreover, MMEF was lower in the MCT positive group (68%±18) compared with (99%±18) in negative MCT group. The mean FEV_1_ was 88%±11 predicted in the asthmatic group and 95%±10 predicted in the non-asthmatic group, both within the normal range. The authors examined the ability of MMEF, R_min_ and FEV_1_ to predict airway hyper-responsiveness to methacholine by producing a receiver operating characteristic (ROC) curve which showed that MMEF had the highest area under the curve (AUC) of 0.87, while R_min_ and FEV_1_ had AUC of 0.85 and 0.78, respectively. R8 was not reported in the ROC curve.

Mousa and Kamal^25^ recruited 25 asthmatic patients and 20 healthy controls (with differences in the mean ages of the groups: asthmatic=45 years and the controls=34 years). Mean BMI did not differ between groups. The severity of the asthmatic group was not reported. IOS was used to assess asthma, with X5 and R5 being reported. R5 was reported in % predicted, but the X5 was reported in absolute values, but did not indicate the unit used. X5 and R5 were different between the two groups (asthma: mean X5 −2.87±1.84 and R5% 245.24±109.18. Healthy controls mean X5 −0.28±0.10 and R5% 109.25±19.40). FEV_1_ was lower in the asthmatic group with a mean of 59.68%±23.73 predicted compared with the healthy controls mean of 89.75%±8.70 predicted.

Koruga *et al*^22^ included 31 male military recruits in Serbia with a previous diagnosis of asthma. Histamine was used to assess bronchial hyperreactivity, recording the dose that decreased FEV_1_ by 20% predicted value (PD20). X5, R5 and Ax was reported at baseline and after PD20. They found that the overall change in FEV_1_ after PD20 was 25.66%, while the R5 and X5 had a change of 66.64±62.91 and 132.18±148.13, respectively. No controls were included in the study.

Similar to Koruga *et al*^22^ and Mousa and Kamal,^25^ Nair *et al*^26^ used a Masterscreen-IOS device, but reported X5 and R5 as % predicted values. Nair *et al* included 82 patients with previous diagnosis of asthma and 61 healthy subjects. The asthma group was older (mean age 49 years vs mean age 28 years in the control group). Weight was not reported in either group. Nineteen per cent of the asthma patients were current or previous smokers, but the controls were all never smokers. All inhaled drugs such as short acting beta-agonists and long acting beta-agonists were withheld before reversibility testing except inhaled corticosteroids. Asthma severity and comorbidities were not reported. Airways reversibility was assessed using 400 μg of salbutamol via a metered dose inhaler and spacer and reported a mean change of 6.34% of FEV_1_ in the asthma group and 2.25% in the healthy controls. The mean percentage of change after administering salbutamol was −33.78±4.43 and −72.93±88.73 in R5 and X5, respectively in the asthma group. In the control group, the mean change was −14.91±2.48 in R5 and 40.09±65.64 in X5.

#### Maximal mid-expiratory flow

Guldent Pasaoglu *et al*^30^ recruited 433 asthmatic patients (mean age 37 years) and 152 patients with COPD (mean age 54 years), aiming to assess differences in clinical and spirometric features of asthma and COPD. 29% of the asthma group and 64% of the COPD group were current smokers. Reversibility was assessed in both groups using 200 μg of salbutamol and defined by an increase of more than 12% and 200 mL of the FEV_1_ value. 62.1% of the asthma group met criteria for reversibility compared with 39.5% in the COPD group. MMEF was the only parameter that was below the normal range in non-smoking asthmatic patients with normal auscultation, suggesting that MMEF was a physiological marker of asthma in non-smoking asymptomatic patients. Although bronchodilator responses were measured, these were not reported.

Son *et al*^31^ conducted a retrospective study of 125 patients with a clinical suspicion of asthma who had undergone an MCT. Patients were stratified into three groups based on their FEV_1_ and MMEF response to MCT. The positive response to MCT was considered if there was a decline of 20% in FEV_1_ and for MMEF, as well. Group 1 included patients with negative MCT tests for both parameters. Group 2 included patients with a negative FEV_1_ and a positive MMEF. Group 3 included patients with positive test to both parameters. The mean ages of the included subjects were 45 years in group 1, 39 years in group 2 and 43 years in group 3. Eight subjects had a previous diagnosis of asthma, three in group 1 and five in group 3. Allergic rhinitis was reported in 34 subjects, 16 of them were positive to MCT in both spirometric indices, therefore included in group 3. Mean baseline MMEF in groups 1 and 2 was 97.67%±3.48 predicted and 95.08%±5.74 predicted, respectively. In group 3, mean MMEF was 70.16%±4.64 predicted. The authors suggested that MMEF may be a more sensitive marker of asthma than FEV_1_ in patients with otherwise normal spirometry results.

### DTA studies

#### Impulse oscillometry

**Figure 3:**
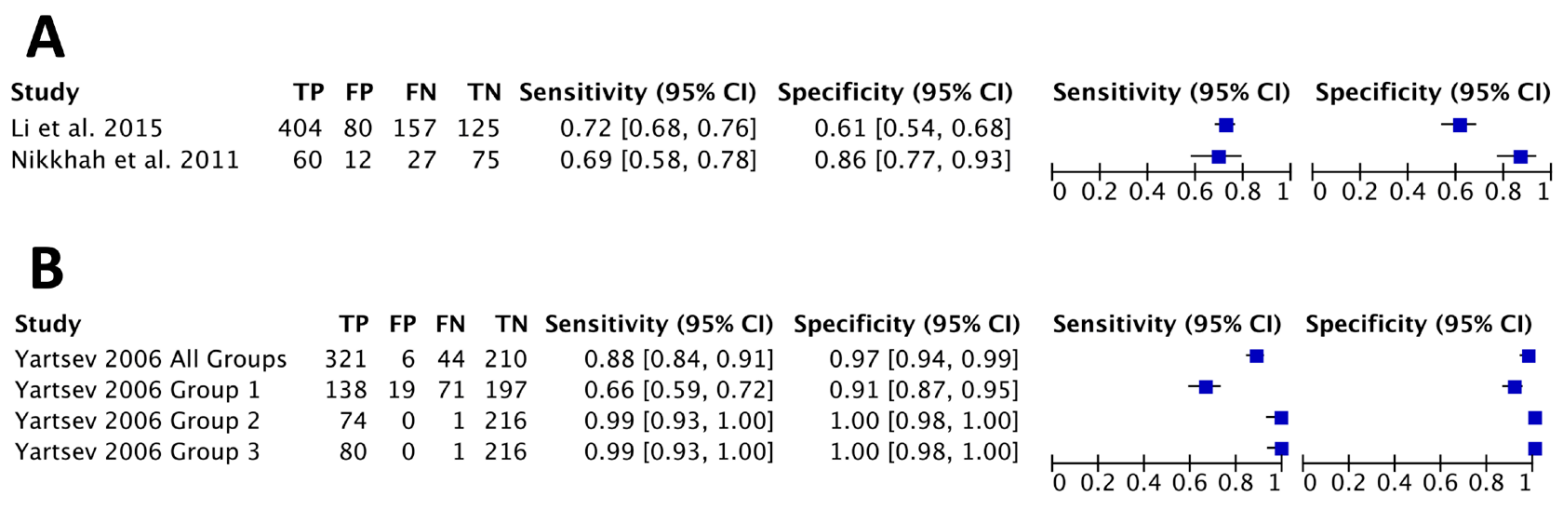
(A) Diagnostic test accuracy (DTA) forest plot of R5. (B) DTA forest plot of maximal mid-expiratory flow. FN, false negative; FP, false positive; TN, true negative; TP, true positive.

Li *et al*^27^ and Nikkhah *et al*^28^ assessed the DTA of IOS for asthma. The majority of participants in Nikkhah *et al* study were women, while Li *et al*^27^ had a majority of male participants. Neither study reported participants’ weight or BMI. Nikkhah *et al* cut-offs were not pre-specified, but were proposed after plotting an ROC curve. Li *et al* did not report a cut-off value. They both studied resistance at 5 Hz, but reactance was studied at different frequencies with Li *et al* at 35 Hz and Nikkhah *et al* at 5 Hz. The sensitivity of R5 was reported as 72% by Li *et al* and 69% by Nikkhah *et al* while specificity reported by Li *et al* at 61% and Nikkhah *et al* at 86%. Reactance had lower sensitivity in both studies. Both studies did not report the asthma severity of the participants or clinical comorbidities and Li *et al* did not report the FEV_1_ results, although they performed bronchodilator response tests on all participants. Figure 3A shows the pooled data of R5 of the two studies.

#### Maximal mid-expiratory flow

There was only one DTA study which used MMEF in asthma compared with controls (Yartsev).^29^ The asthma group was older than the control group. Both the asthma and control groups had a majority of female participants. Asthma severity and comorbidities of participants were not reported. The author stratified asthma patients into three groups based on the baseline FEV_1_. Group 1 included participants with FEV_1_ of >80% predicted value, group 2 with FEV_1_ 60%–80% predicted and group 3 with FEV_1_ 25%–60% predicted. In MMEF tests, the cut-off used was 90% predicted in group 1, 70% predicted in group 2 and 50% predicted in group 3. The DTA of MMEF in group 1 was a sensitivity of 66% and specificity of 91%. Identical results were found in groups 2 and 3 with a sensitivity of 99% and specificity of 100%. The accuracy of MMEF was assessed on all groups with a cut-off value of 70% showed a sensitivity of 88% and specificity 97%. Using FEV_1_, cut-off was set at 120% predicted in group 1, 90% predicted in group 2, 70% predicted in group 3. The DTA of FEV_1_ in group 1 was a sensitivity of 77% and specificity of 65%. In groups 2 and 3, identical sensitivity of 100% and specificity of 100% was reported. All groups were assessed for accuracy using FEV_1_, with a 70% predicted cut-off, showing a sensitivity of 92% and specificity of 88%. DTA data were pooled into the forest plot shown in figure 3B.

### Synthesis of results

**Figure 4:**
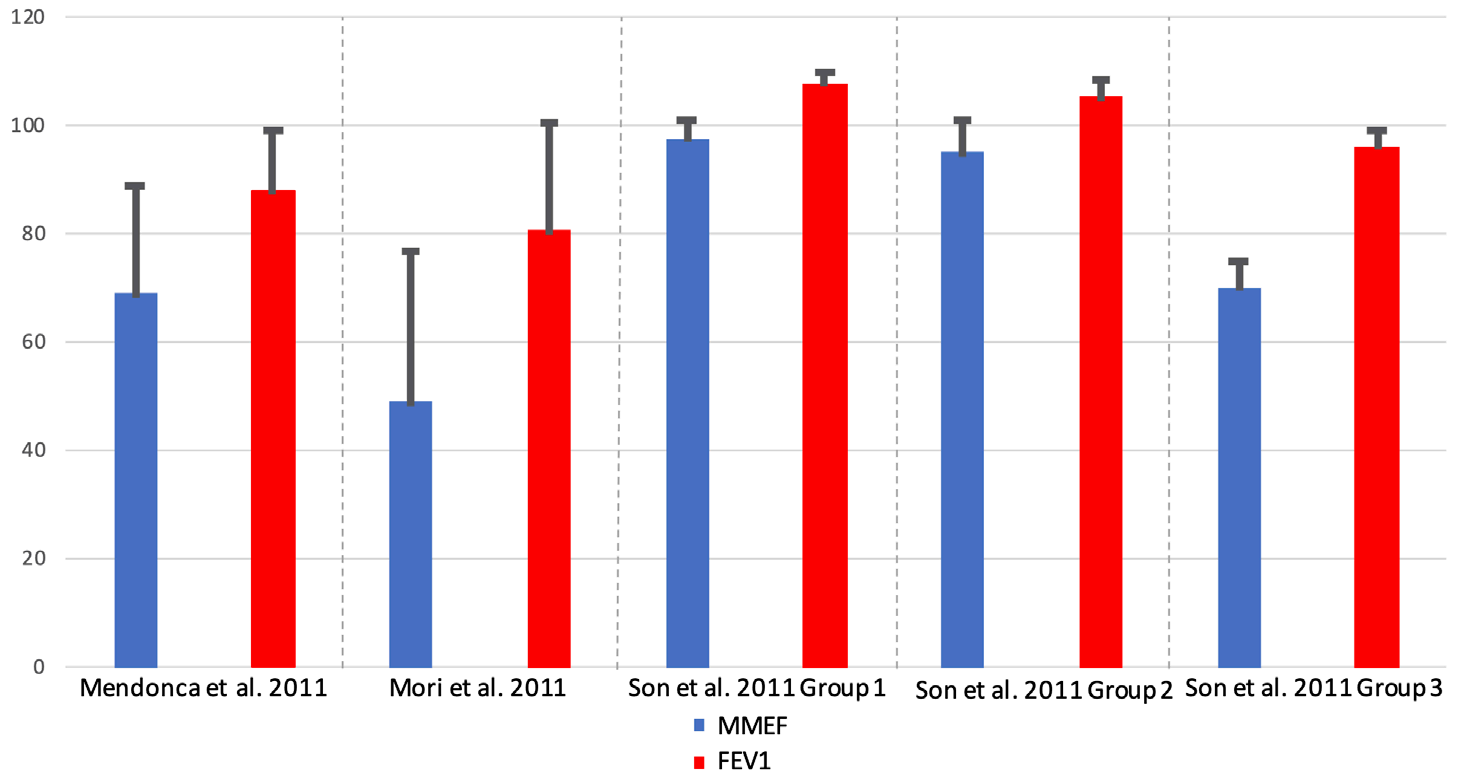
Comparison of % predicted of MMEF to FEV_1_ in asthmatic patients. FEV_1_, forced expiratory volume in 1 s; MMEF, maximal mid-expiratory flow.

**Figure 5:**
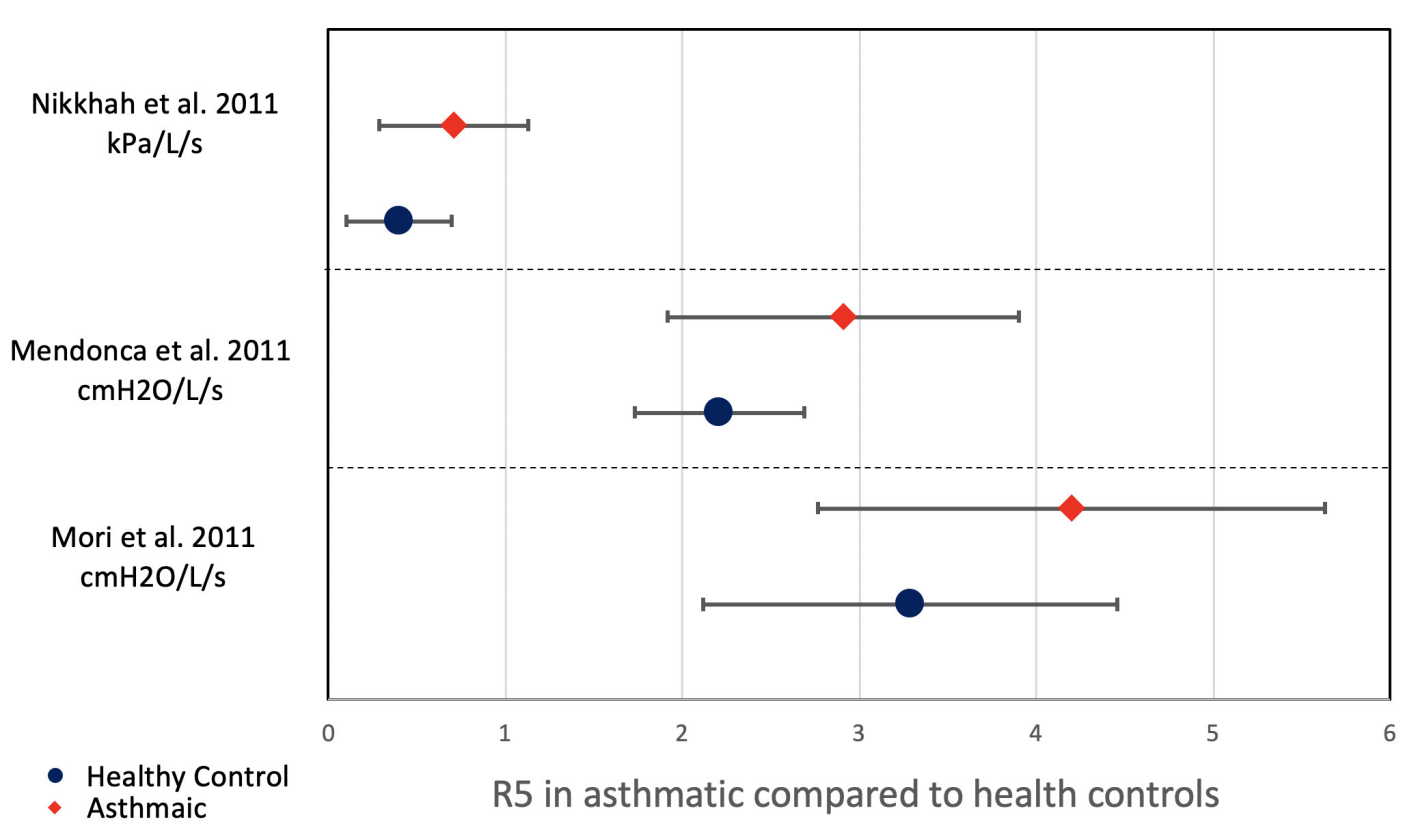
Comparison of R5 between asthmatic patients to healthy controls.

Small airways function in asthma were found to be different when compared with healthy controls. The % predicted MMEF value appeared consistently lower than the % predicted FEV_1_, as shown in figure 4. In oscillometry, R5 was also found to be consistently higher in asthmatic when compared with healthy controls as shown in figure 5. These results highlight the presence of small airways limitation in asthmatic patients with heterogeneous characteristics including age, ethnicity and weight.

### Risk of bias across studies

There were some concerns of bias in regard to reporting outcomes. Kamal and Mousa^25^ reported R5 as the % predicted value while X5 was reported without a unit of measurement. Iartsev^29^ did not report how the cut-offs were determined or how subjects were recruited.

## Discussion

To the authors’ knowledge, this is the first systematic review to assess the use of physiological tests of small airways function in the diagnosis of asthma. Previous work has suggested that SAD is associated with asthma and that the prevalence of SAD increases with the severity of asthma.^32 33^ This review suggests that most published studies of small airways function tests in asthma are heterogeneous, of varying methodological quality and have primarily identified SAD rather than using measures of small airways to diagnose asthma. No studies reported the severity of asthma in the participants and participants groups were often poorly matched or characterised in terms of other comorbidities and weight.

This review focuses on MMEF and oscillometry and does not explore all potential measures to assess small airways function. MMEF and oscillometry were chosen as these represented the most commonly cited small airways measures. The clinical utility of oscillometry techniques has been described in asthma and other lung conditions such as interstitial lung diseases and COPD.^19^ Oscillometry has been suggested as a useful tool in diagnosing asthma in children.^34^ However, there remains a lack of universal reference ranges, especially in adults. Height^23^ and sex^35^ appear to alter values. Oostveen *et al*^35^ conducted a multicentre study on healthy subjects in an effort to produce reference ranges for oscillometry in adults, but only one ethnicity was studied. Another study was also conducted in Japan to establish reference ranges for Japanese adults.^36^ Understanding and interpreting oscillometry remains challenging. In this review, it was unclear if oscillometry studies provided the most robust measure of small airways function. The R5–R20 (often referred to as resistance of the small airways) was only reported by Mori *et al*.^24^ Airways reversibility, a hallmark of asthma, was only assessed using oscillometry by Nair *et al*^26^ and, here, the mean percentage change in the FEV_1_ in the asthma group was 6.34%, which is less than the standard reversibility change of 12%.

The MMEF is an effort-dependent test and guidelines for reproducibility of the manoeuvre is based on FVC and FEV_1_.^37^ In all the included articles that studies MMEF, the % predicted of MMEF was found to be lower in asthmatic groups compared with control groups. Moreover, the % predicted value of MMEF was lower than the % predicted FEV_1_ in the asthmatic group, suggesting that small airways limitation might be an early marker of airways obstruction. The potential utility of MMEF in early disease was described in one study of patients with alpha-1 antitrypsin deficiency, where an MMEF less than 80% predicted, with a normal FEV_1_/FVC ratio, was associated with increased respiratory symptoms and a faster decline in FEV_1_ compared with those with an MMEF of 80% or greater and normal spirometry, suggesting a role for MMEF in early disease monitoring.^38^

There are significant limitations to the evidence base described in this review including study heterogeneity, poor patient characterisation and differences in reported values. Not all tests of small airways function have been assessed in asthma (eg, MBW). There are no universally accepted predicted values for oscillometry, especially in adults, making the interpretation of the results more difficult. Oscillation techniques produce many parameters in both inspiratory and expiratory phases and the differences in reported values limits comparisons between studies. MMEF was not corrected for FVC in any study, and this is a limitation as MMEF is a timed/flow measurement and FVC exhalation curve changes may affect the results.^39^ Nevertheless, most studies provide at least some signal of SAD in asthma suggesting these indices could be helpful in diagnosing and monitoring asthma. To take this field forward, further research is needed. This should include standardising the assessment of small airways tests (although different tests may have greater or lesser utility in different diseases) and forming normal reference ranges to aid interpretation. Studies in asthma need to predefine how asthma was diagnosed, and report clearly which small airways tests have been measured, by what device, what units are reported and what would be considered an abnormal result or clinically meaningful change in a specified value.

## Conclusion

Physiological tests of small airways function are feasible in diagnosing asthma and have been shown to be altered in asthma when compared with healthy adults. However, a lack of robust reference ranges and the heterogeneity of approach complicate their use.

Further studies are needed to assess small airways function in asthma, especially in early disease. Larger studies are needed to assess the impact of demographic characteristics and comorbidities such as obesity or allergic rhinitis. This systematic review of current literature suggests these tests may have promise as part of the future diagnostic criteria of asthma, but more work is needed before they can be embedded into clinical care.
