# Supplementary material for: Physiological tests of small airways function in diagnosing asthma: a systematic review
Source: BMJ Open Respir Res. 2020 Dec 21;7(1):e000770. doi: 10.1136/bmjresp-2020-000770 (PMC7754643; doi:10.1136/bmjresp-2020-000770)
Supplement: Supplementary data [file bmjresp-2020-000770supp002.pdf]

BMJ Publishing Group Limited (BMJ) disclaims all liability and responsibility arising from any reliance placed on this supplemental material which has been supplied by the author(s)


# PICO Table

**Population**

- Adults >18 years old with asthma or clinical suspicion of asthma

**Intervention**

- Physiological tests small airways function

**Comparator**

- $FEV_1$

**Outcome**

- Diagnosis of asthma

PICO chart with details of study selection criteria.
