# Supplementary material for: Physiological tests of small airways function in diagnosing asthma: a systematic review
Source: BMJ Open Respir Res. 2020 Dec 21;7(1):e000770. doi: 10.1136/bmjresp-2020-000770 (PMC7754643; doi:10.1136/bmjresp-2020-000770)
Supplement: Supplementary data [file bmjresp-2020-000770supp003.pdf]

Medline:

1. exp Asthma/
2. exp Bronchial Spasm/
3. exp Bronchial Hyperreactivity/ or exp Respiratory Hypersensitivity/
4. exp Airway Resistance/ or exp Bronchoconstriction/
5. airway reactivity.ab,ti.
6. exp Respiratory Function Tests/
7. small airway*.ab,ti.
8. distal airway*.ti,ab.
9. MMEF.mp.
10. fef25*.mp.
11. FOT.mp.
12. Forced Oscillatory.mp.
13. Impulse Oscillatory.mp. or exp Oscillometry/
14. Impulse Oscillatory.mp.
15. Impulse Oscillimatry.mp.
16. Impulse Oscillation.mp.
17. (IOS and airway*).mp.
18. exp Nitric Oxide/ and asthma.mp.
19. Fractional expired nitric oxide.ti,ab.
20. Fractional exhaled nitric oxide.ti,ab.
21. FeNO.ti,ab.
22. (FeNO and asthma).ti,ab.
23. multiple breath washout.mp.
24. MBW.ti,ab.
25. MBW.ti,ab. and airway*.mp.
26. exp Spirometry/
27. (Reversibility and asthma).ti,ab.
28. exp Respiratory Function Tests/
29. exp Peak Expiratory Flow Rate/
30. exp Asthma/
31. 6 or 7 or 8 or 9 or 10 or 11 or 12 or 13 or 14 or 15 or 16 or 17 or 18 or 19 or 20 or 21 or 22 or 23 or 24 or 25
32. 26 or 27 or 28 or 29
33. adult*.mp.
34. 1 or 2 or 3 or 4 or 5
35. 30 and 31 and 32 and 33 and 34
36. Diagnos*.ti,ab.
37. 35 and 36

Results: 1856

EMBASE:

1. exp asthma/
2. exp bronchospasm/
3. exp bronchus hyperreactivity/
4. Respiratory Hypersensitivity.mp.
5. exp airway resistance/
6. exp bronchoconstriction/
7. airway reactivity.ab,ti.
8. 1 or 2 or 3 or 4 or 5 or 6 or 7
9. exp lung function test/
10. small airway*.ab,ti.
11. distal airway*.ti,ab.
12. MMEF.mp.
13. fef25*.mp.
14. FOT.mp.
15. Forced Oscillatory.mp.
16. Impulse Oscillatory.mp. or exp Oscillometry/
17. Impulse Oscillatory.mp.
18. Impulse Oscillimetry.mp.
19. Impulse Oscillation.mp.
20. (IOS and airway*).mp.
21. exp Nitric Oxide/ and asthma.mp.
22. Fractional expired nitric oxide.ti,ab.
23. Fractional exhaled nitric oxide.ti,ab.
24. (FeNO and asthma).ti,ab.
25. multiple breath washout.ti,ab.
26. MBW.ti,ab. and airway*.mp.
27. exp spirometry/
28. (Reversibility and asthma).ti,ab.
29. exp lung function test/
30. exp peak expiratory flow/
31. exp asthma/
32. adult*.mp.
33. diagnos*.ti,ab.
34. 9 or 10 or 11 or 12 or 13 or 14 or 15 or 16 or 17 or 18 or 19 or 20 or 21 or 22 or 23 or 24 or 25 or 26
35. 27 or 28 or 29 or 30
36. 8 and 31 and 32 and 33 and 34 and 35

Results: 2553

Web Of Science:

TS=(Asthma OR bronchospasm OR bronchus hyperreactivity OR Respiratory Hypersensitivity OR airway resistance OR bronchoconstriction OR airway reactivity)

AND

TS=(lung function test OR small airway* OR distal airway* OR Pulmonary function OR PFT OR MMEF OR fef25* OR FOT OR Forced Oscillatory OR Impulse Oscillatory OR Oscillometry OR Impulse Oscillimetry OR Impulse Oscillation OR IOS OR Nitric Oxide ORFractional expired nitric oxide OR Fractional exhaled nitric oxide OR FeNO OR multiple breath washout OR MBW)

AND

TS=(Spirometry OR Reversibility OR lung function test* OR peak expiratory flow OR PEF*)

AND

TS=(Adult*)

AND

TS=(Diagnos*)

Up to 5 years = 975

CENTRAL (Cochrane)

#1 Asthma OR bronchospasm OR bronchus hyperreactivity OR Respiratory Hypersensitivity OR airway resistance OR bronchoconstriction OR airway reactivity
#2 lung function test OR small airway* OR distal airway* OR Pulmonary function OR PFT OR MMEF OR fef25* OR FOT OR Forced Oscillatory OR Impulse Oscillatory OR Oscillometry OR Impulse Oscillimetry OR Impulse Oscillation OR IOS OR Nitric Oxide ORFractional expired nitric oxide OR Fractional exhaled nitric oxide OR FeNO OR multiple breath washout OR MBW
#3 Spirometry OR Reversibility OR lung function test* OR peak expiratory flow OR PEF*
#4 adult*
#5 diagnos*
#6 #1 AND #2 AND #3 AND #4 AND #5

Results: 813

CINAHL:

S1 (MH "Asthma+")
S2 (MM "Bronchial Spasm")
S3 TI bronchospasm OR AB bronchospasm
S4 "bronchus hyperreactivity"
S5 "bronchus hyperreactivity"
S6 TI bronchus hyperreactivity OR AB bronchus hyperreactivity
S7 TI bronchus hyperreactivity OR AB bronchus hyperreactivity
S8 (MH "Respiratory Hypersensitivity+")
S9 (MM "Airway Resistance")
S10 TI airway resistance OR AB airway resistance
S11 (MM "Bronchoconstriction")
S12 "airway reactivity"
S13 S1 OR S2 OR S3 OR S4 OR S5 OR S6 OR S7 OR S8 OR S9 OR S10 OR S11 OR S12
S14 (MH "Respiratory Function Tests+")
S15 TI small airway* OR AB small airway*
S16 TI distal airway* OR AB distal airway*
S17 TI MMEF OR AB MMEF
S18 TI fef25* OR AB fef25*
S19 TI FOT OR AB FOT
S20 TI MMEF OR AB MMEF AND airway*
S21 TI Forced Oscillatory OR AB Forced Oscillatory
S22 TI Impulse Oscillimetry OR AB Impulse Oscillimetry
S23 TI Impulse Oscillimetry OR AB Impulse Oscillimetry
S24 TI Impulse Oscillation OR AB Impulse Oscillation
S25 TI (IOS and airway*) OR AB (IOS and airway*)
S26 AB (Nitric Oxide AND asthma) OR TI ((Nitric Oxide AND asthma)
S27 AB (feno AND asthma) OR TI (feno AND asthma)
S28 TI multiple breath washout OR AB multiple breath washout
S29 "MBW"
S30 S14 OR S15 OR S16 OR S17 OR S18 OR S19 OR S20 OR S21 OR S22 OR S23 OR S24 OR S25 OR S26 OR S27 OR S28 OR S29
S31 (MM "Spirometry") OR (MM "Airway Resistance") OR (MH "Respiratory Airflow+") OR (MH "Forced Expiratory Flow Rates+") OR (MM "Peak Expiratory Flow Rate")
S32 "diagnos*"
S33 S1 AND S13 AND S30 AND S31 AND S32
S34 (MH "Asthma+")
S35 (MM "Bronchial Spasm")
S36 TI bronchospasm OR AB bronchospasm
S37 "bronchus hyperreactivity"
S38 "bronchus hyperreactivity"
S39 TI bronchus hyperreactivity OR AB bronchus hyperreactivity
S40 TI bronchus hyperreactivity OR AB bronchus hyperreactivity
S41 (MH "Respiratory Hypersensitivity+")
S42 (MM "Airway Resistance")

S43 TI airway resistance OR AB airway resistance
S44 (MM "Bronchoconstriction")
S45 "airway reactivity"
S46 S34 OR S35 OR S36 OR S37 OR S38 OR S39 OR S40 OR S41 OR S42 OR S43 OR S44 OR S45
S47 (MH "Respiratory Function Tests+")
S48 TI small airway* OR AB small airway*
S49 TI distal airway* OR AB distal airway*
S50 TI MMEF OR AB MMEF
S51 TI fef25* OR AB fef25*
S52 TI FOT OR AB FOT
S53 TI MMEF OR AB MMEF AND airway*
S54 TI Forced Oscillatory OR AB Forced Oscillatory
S55 TI Impulse Oscillimetry OR AB Impulse Oscillimetry
S56 TI Impulse Oscillimetry OR AB Impulse Oscillimetry
S57 TI Impulse Oscillation OR AB Impulse Oscillation
S58 TI (IOS and airway*) OR AB (IOS and airway*)
S59 AB (Nitric Oxide AND asthma) OR TI ((Nitric Oxide AND asthma)
S60 AB (feno AND asthma) OR TI (feno AND asthma)
S61 TI multiple breath washout OR AB multiple breath washout
S62 "MBW"
S63 S47 OR S48 OR S49 OR S50 OR S51 OR S52 OR S53 OR S54 OR S55 OR S56 OR S57 OR S58 OR S59 OR S60 OR S61 OR S62

Results: 875

Total Before duplicates removal = 7072
