# Supplementary material for: Physiological tests of small airways function in diagnosing asthma: a systematic review
Source: BMJ Open Respir Res. 2020 Dec 21;7(1):e000770. doi: 10.1136/bmjresp-2020-000770 (PMC7754643; doi:10.1136/bmjresp-2020-000770)
Supplement: Supplementary data [file bmjresp-2020-000770supp004.pdf]

Excluded Articles.

Reason of Exclusion	Citations Numbers
A. Wrong Outcome	1-264
B. Wrong Population	265-304
C. Wrong Study Design	305-368
D. Abstracts Only	369-446
E. Review articles	447-449
F. Duplicates	450-457

A. (1-264)
B. (265-304)
C. (305-368)
D. (369-446)
E. (447-449)
F. (450-457)

1. Aamodt T, Lien JT, Haaversen O, et al. The effect of slow-release and microcrystalline theophylline preparations in asthmatic adults. A randomised double-blind, crossover comparison of Nuelin Depot, Nuelin and placebo. Tidsskrift for den Norske laegeforening. 1983;103(17):1397-9+402.
2. Akamatsu T, Shirai T, Shimoda Y, et al. Forced oscillation technique as a predictor of FEV1 improvement in asthma. Respiratory Physiology & Neurobiology. 2017;236:78-83.
3. Alberts WM, Ferris MC, Brooks SM, et al. The FEF25-75% and the clinical diagnosis of asthma. Ann Allergy. 1994;73(3):221-5.
4. Ali Z, Nilas L, Ulrik CS. Determinants of low risk of asthma exacerbation during pregnancy. Clin Exp Allergy. 2018;48(1):23-8.
5. Al-Mutairi SS, Sharma PN, Al-Alawi A, et al. Impulse oscillometry: an alternative modality to the conventional pulmonary function test to categorise obstructive pulmonary disorders. Clin Exp Med. 2007;7(2):56-64.
6. Al-Shamkhi N, Alving K, Dahlen SE, et al. Important non-disease-related determinants of exhaled nitric oxide levels in mild asthma - results from the Swedish GA(2) LEN study. Clin Exp Allergy. 2016;46(9):1185-93.
7. Alvarez-Puebla MJ, Olaguibel Rivera JM, Almudevar E, et al. Cutoff point for exhaled nitric oxide corresponding to 3% sputum eosinophils. J Investig Allergol Clin Immunol. 2015;25(2):107-11.
8. Anderson SD, Charlton B, Weiler JM, et al. Comparison of mannitol and methacholine to predict exercise-induced bronchoconstriction and a clinical diagnosis of asthma. Respir Res. 2009;10:4.
9. Badnjevic A, Cifrek M, Koruga D, et al. Neuro-fuzzy classification of asthma and chronic obstructive pulmonary disease. BMC Med Inform Decis Mak. 2015;15 Suppl 3:S1.
10. Bag R, Bandi V, Fromm RE, Jr., et al. The effect of heliox-driven bronchodilator aerosol therapy on pulmonary function tests in patients with asthma. J Asthma. 2002;39(7):659-65.
11. Bajera I, Maleszka P, From S. [Analysis of ventilation parameters before and after fiber optic bronchoscopy in patients with atopic bronchial asthma and chronic obstructive pulmonary diseases]. Pol Merkur Lekarski. 1997;3(16):171-3.
12. Baltieri L, Martins LC, Cazzo E, et al. Analysis of quality of life among asthmatic individuals with obesity and its relationship with pulmonary function: cross-sectional study. Sao Paulo Medical Journal. 2017;135(4):332-8.
13. Bao WP, Zhang X, Lv CJ, et al. The Value of Fractional Exhaled Nitric Oxide and Forced Mid-Expiratory Flow as Predictive Markers of Bronchial Hyperresponsiveness in Adults with Chronic Cough. J Allergy Clin Immunol-Pract. 2018;6(4):1313-20.

14. Baptist AP, Ross JA, Clark NM. Older adults with asthma: does age of asthma onset make a difference? J Asthma. 2013;50(8):836-41.
15. Beckett WS, Marenberg ME, Pace PE. Repeated methacholine challenge produces tolerance in normal but not in asthmatic subjects. Chest. 1992;102(3):775-9.
16. Bennett GH, Carpenter L, Hao W, et al. Risk factors and clinical outcomes associated with fixed airflow obstruction in older adults with asthma. Ann Allergy Asthma Immunol. 2018;120(2):164-8 e1.
17. Berdel D, Holle JP, Hartmann V, et al. [Significance of measurement of the oscillatory resistance to respiration in children. (author's transl)]. Klin Padiatr. 1981;193(2):73-6.
18. Beretta C, Rifflart C, Evrard G, et al. Assessment of eosinophilic airway inflammation as a contribution to the diagnosis of occupational asthma. Allergy. 2018;73(1):206-13.
19. Berkman N, Avital A, Breuer R, et al. Exhaled nitric oxide in the diagnosis of asthma: comparison with bronchial provocation tests. Thorax. 2005;60(5):383-8.
20. Bernstein JA, Davis B, Alvarez-Puebla MJ, et al. Is exhaled nitric oxide a useful adjunctive test for assessing asthma? J Asthma. 2009;46(9):955-60.
21. Berry M, Hargadon B, Morgan A, et al. Alveolar nitric oxide in adults with asthma: evidence of distal lung inflammation in refractory asthma. Eur Respir J. 2005;25(6):986-91.
22. Bishopp A, Sathyamurthy R, Manney S, et al. Biomarkers of oxidative stress and antioxidants in severe asthma: A Prospective Case-Control Study. Ann Allergy Asthma Immunol. 2017;118(4):445-51.
23. Bjerregaard A, Laing IA, Backer V, et al. High fractional exhaled nitric oxide and sputum eosinophils are associated with an increased risk of future virus-induced exacerbations: A prospective cohort study. Clin Exp Allergy. 2017;47(8):1007-13.
24. Bobolea I, Barranco P, Del Pozo V, et al. Sputum periostin in patients with different severe asthma phenotypes. Allergy. 2015;70(5):540-6.
25. Bokov P, Martin C, Graba S, et al. Bronchodilator Response Assessment of the Small Airways Obstructive Pattern. Open Respir Med J. 2017;11:47-53.
26. Borodin Iu P, Dorogova OA. [Use of the acetylcholine inhalation test in the complex diagnosis of chronic asthmatic bronchitis and bronchial asthma]. Voen Med Zh. 1984(11):55-6.
27. Brindicci C, Ito K, Barnes PJ, et al. Differential flow analysis of exhaled nitric oxide in patients with asthma of differing severity. Chest. 2007;131(5):1353-62.
28. Broekhuizen BD, Sachs AP, Hoes AW, et al. Undetected chronic obstructive pulmonary disease and asthma in people over 50 years with persistent cough. Br J Gen Pract. 2010;60(576):489-94.
29. Brown RH, Togias A. Measurement of intraindividual airway tone heterogeneity and its importance in asthma. Journal of applied physiology (Bethesda, Md : 1985). 2016;121(1):223-32.
30. Bulac S, Cimrin A, Ellidokuz H. The effect of beclometasone dipropionate/formoterol extra-fine fixed combination on the peripheral airway inflammation in controlled asthma. J Aerosol Med Pulm Drug Deliv. 2015;28(2):82-7.
31. Burgess G, Boyce M, Jones M, et al. Randomized study of the safety and pharmacodynamics of inhaled interleukin-13 monoclonal antibody fragment VR942. EBioMedicine. 2018;35:67-75.
32. Busse WW, Holgate ST, Wenzel SW, et al. Biomarker Profiles in Asthma With High vs Low Airway Reversibility and Poor Disease Control. Chest. 2015;148(6):1489-96.
33. Butzko RP, Sotolongo AM, Helmer DA, et al. Forced oscillation technique in veterans with preserved spirometry and chronic respiratory symptoms. Respir Physiolo Neurobiol. 2019;260:8-16.

34. Byrne AL, Marais BJ, Mitnick CD, et al. Asthma and atopy prevalence are not reduced among former tuberculosis patients compared with controls in Lima, Peru. BMC Pulm Med.19(40).
35. Calciano L, Portas L, Corsico AG, et al. Biomarkers related to respiratory symptoms and lung function in adults with asthma. J Breath Res. 2018;12(2):026012.
36. Carpio C, Villasante C, Galera R, et al. Systemic inflammation and higher perception of dyspnea mimicking asthma in obese subjects. J Allergy Clin Immunol. 2016;137(3):718-26 e4.
37. Chambers L, Finch J, Edwards K, et al. Effects of personal air pollution exposure on asthma symptoms, lung function and airway inflammation. Clin Exp Allergy. 2018;48(7):798-805.
38. Chen CZ, Lin CC, Lee CH, et al. Small airways obstruction syndrome in clinical practice. Respirology. 2009;14(3):393-8.
39. Chen FJ, Huang XY, Liu YL, et al. Importance of fractional exhaled nitric oxide in the differentiation of asthma-COPD overlap syndrome, asthma, and COPD. Int J Chron Obstruct Pulmon Dis. 2016;11:2385-90.
40. Cioe K, Biondi BE, Easly R, et al. A systematic review of patients' and providers' perspectives of medications for treatment of opioid use disorder. J Subst Abuse Treat. 2020;119(4):108146.
41. Cipr, i G, Schiavetti I, et al. Symptom perception and asthma control. Postgraduate Medical Journal. 2015;127(7):738-43.
42. Connolly MJ, Kelly C, Walters EH, et al. An assessment of methacholine inhalation tests in elderly asthmatics. Age Ageing. 1988;17(2):123-8.
43. Coop C, Hagan LL, Dice JP. Exhaled breath condensate pH in the evaluation of asthma. Allergy Asthma Proc. 2008;29(1):51-4.
44. Cordeiro D, Rudolphus A, Snoey E, et al. Utility of nitric oxide for the diagnosis of asthma in an allergy clinic population. Allergy Asthma Proc. 2011;32(2):119-26.
45. Crespo A, Giner J, Torrejon M, et al. Clinical and inflammatory features of asthma with dissociation between fractional exhaled nitric oxide and eosinophils in induced sputum. J Asthma. 2016;53(5):459-64.
46. Dal Negro RW, Tognella S, Micheletto C. Pharmacokinetics of the effect of nebivolol 5mg on airway patency in patients with mild to moderate bronchial asthma and arterial hypertension: a randomised, placebo-controlled study. Clinical Drug Investigation. 2002;22(3):197-204.
47. Dales RE, Cakmak S. Is residential ambient air limonene associated with asthma? Findings from the Canadian Health Measures Survey.244:966-70.
48. David M, Chhajed PN, Tamm M, et al. Diagnostic tests for asthma in firefighters. Chest. 2007;131(6):1760-7.
49. Decramer M, Janssens W, Derom E, et al. Contribution of four common pulmonary function tests to diagnosis of patients with respiratory symptoms: a prospective cohort study. Lancet Respir Med. 2013;1(9):705-13.
50. Demange V, Bohadana A, Massin N, et al. Exhaled nitric oxide and airway hyperresponsiveness in workers: a preliminary study in lifeguards. BMC Pulm Med. 2009;9:53.
51. Deng DD, Zhou AY, Shuang QC, et al. [The value of fractionated exhaled nitric oxide in the diagnosis of asthma-chronic obstructive pulmonary disease overlap syndrome]. Zhonghua Jie He He Hu Xi Za Zhi. 2017;40(2):98-101.
52. Descatha A, Fromageot C, Ameille J, et al. Is forced oscillation technique useful in the diagnosis of occupational asthma? J Occup Environ Med. 2005;47(8):847-53.

53. Dickinson JW, Whyte GP, McConnell AK, et al. Mid-expiratory flow versus FEV1 measurements in the diagnosis of exercise induced asthma in elite athletes. Thorax. 2006;61(2):111-4.
54. Dupont LJ, Demedts MG, Verleden GM. Prospective evaluation of the validity of exhaled nitric oxide for the diagnosis of asthma. Chest. 2003;123(3):751-6.
55. Dweik RA, Sorkness RL, Wenzel S, et al. Use of Exhaled Nitric Oxide Measurement to Identify a Reactive, at-Risk Phenotype among Patients with Asthma. American Journal of Respiratory and Critical Care Medicine. 2010;181(10):1033-41.
56. Dziedziczko A, Gniazdowski R. [Occurrence of early and late asthmatic reaction after provocation with antigen and the status of pulmonary ventilation in patients with hay fever after the pollination season]. Pol Tyg Lek. 1992;47(34-35):742-4.
57. Ebner L, He M, Virgincar RS, et al. Hyperpolarized (129)Xenon Magnetic Resonance Imaging to Quantify Regional Ventilation Differences in Mild to Moderate Asthma A Prospective Comparison Between Semiautomated Ventilation Defect Percentage Calculation and Pulmonary Function Tests. Investigative Radiology. 2017;52(2):120-7.
58. Ekroos H, Rouhos A, Pallasaho P, et al. Equally elevated concentrations of exhaled nitric oxide in nonatopic and low-sensitized atopic asthmatics. Respir Med. 2009;103(1):152-8.
59. Elbouhy MS, Fattah EBA, Hashem AEM. Effect of inhaled corticosteroids on small airways in asthmatics using impulse oscillometry. Egyptian Journal of Chest Diseases and Tuberculosis. 2018;67(3):214-20.
60. ElHalawani SM, Ly NT, Mahon RT, et al. Exhaled nitric oxide as a predictor of exercise-induced bronchoconstriction. Chest. 2003;124(2):639-43.
61. El-Khatib MF, Jamaleddine G, Kanj N, et al. Effect of heliox- and air-driven nebulized bronchodilator therapy on lung function in patients with asthma. Lung. 2014;192(3):377-83.
62. Engel J, van Kampen V, Lotz A, et al. An increase of fractional exhaled nitric oxide after specific inhalation challenge is highly predictive of occupational asthma. International Archives of Occupational and Environmental Health. 2018;91(7):799-809.
63. Fairbairn DK. Delayed Birth of the Second Twin. Ind Med Gaz. 1932;67(7):389-90.
64. Farha S, Asosingh K, Laskowski D, et al. Effects of the menstrual cycle on lung function variables in women with asthma. Am J Respir Crit Care Med. 2009;180(4):304-10.
65. Farrow CE, Salome CM, Harris BE, et al. Peripheral ventilation heterogeneity determines the extent of bronchoconstriction in asthma. Journal of applied physiology (Bethesda, Md : 1985). 2017;123(5):1188-94.
66. Ferrazzoni S, Scarpa MC, Guarnieri G, et al. Exhaled nitric oxide and breath condensate ph in asthmatic reactions induced by isocyanates. Chest. 2009;136(1):155-62.
67. Fingleton J, Huang K, Weatherall M, et al. Phenotypes of symptomatic airways disease in China and New Zealand. Eur Respir J. 2017;50(6).
68. Fortuna AM, Feixas T, Gonzalez M, et al. Diagnostic utility of inflammatory biomarkers in asthma: exhaled nitric oxide and induced sputum eosinophil count. Respir Med. 2007;101(11):2416-21.
69. Fukuhara A, Saito J, Sato S, et al. Validation study of asthma screening criteria based on subjective symptoms and fractional exhaled nitric oxide. Ann Allergy Asthma Immunol. 2011;107(6):480-6.
70. Fukuhara M, Tsuburai T, Nakamura Y, et al. The Fraction of Exhaled Nitric Oxide (Feno) and Forced Oscillation Technique (Fot) Can Predict Bronchial Hyperresponsiveness against Acetylcholine in Treated Asthmatics. Arerugi. 2017;66(1):42-9.
71. Galvao Lucas J, Palma Carlos AG, Palma Carlos ML. [Comparative study of the instantaneous maximum expiratory flow and of the maximum secondary expiratory volume in the functional diagnosis of asthmatic patients]. Acta Allergol. 1967;22(5):378-86.

72. Gao J, Chen ZC, Jie X, et al. Both fractional exhaled nitric oxide and sputum eosinophil were associated with uncontrolled asthma. Journal of Asthma and Allergy. 2018;11:73-9.
73. Gao J, Wu F. Association between fractional exhaled nitric oxide, sputum induction and peripheral blood eosinophil in uncontrolled asthma. Allergy Asthma Clin Immunol. 2018;14.
74. Gelb AF, Taylor CF, Nussbaum E, et al. Alveolar and airway sites of nitric oxide inflammation in treated asthma. Am J Respir Crit Care Med. 2004;170(7):737-41.
75. Girdhar A, Kumar V, Singh A, et al. Systemic inflammation and its response to treatment in patients with asthma. Respir Care. 2011;56(6):800-5.
76. Gonem S, Hardy S, Buhl N, et al. Characterization of acinar airspace involvement in asthmatic patients by using inert gas washout and hyperpolarized (3)helium magnetic resonance. J Allergy Clin Immunol. 2016;137(2):417-25.
77. Greenspon LW, Gracely E. A discriminant analysis applied to methacholine bronchoprovocation testing improves classification of patients as normal, asthma, or COPD. Chest. 1992;102(5):1419-25.
78. Gronke L, Kanniess F, Holz O, et al. The relationship between airway hyper-responsiveness, markers of inflammation and lung function depends on the duration of the asthmatic disease. Clin Exp Allergy. 2002;32(1):57-63.
79. Guan WJ, Zheng JP, Gao Y, et al. Impulse oscillometry for leukotriene D4 inhalation challenge in asthma. Respir Care. 2013;58(12):2120-6.
80. Gulmez SE, Celik G, Misirligil Z, et al. Dipyrone improves small airway function in asthmatic patients with moderate obstruction. J Investig Allergol Clin Immunol. 2007;17(4):242-8.
81. Gumeniuk NI, Berezhnaia IV, Tsygankova LM. [The characteristics of postloading bronchospasm in bronchial asthma patients]. Vrach Delo. 1990(12):39-42.
82. Hara J, Fujimura M, Myou S, et al. Sputum eosinophilia, airway hyperresponsiveness and airway narrowing in young adults with former asthma. Allergology international : official journal of the Japanese Society of Allergology. 2008;57(3):211-7.
83. Hardaker KM, Downie SR, Kermode JA, et al. Predictors of Airway Hyperresponsiveness Differ Between Old and Young Patients With Asthma. Chest. 2011;139(6):1395-401.
84. Harnan S, Essat M, Gomersall T, et al. Exhaled Nitric Oxide for the Diagnosis of Asthma in Adults and Children: A Systematic Review. Value in Health. 2015;18(7):A345-A.
85. He L, Wei M, Luo J, et al. Re-evaluation of the diagnostic value of fractional exhaled nitric oxide & its impact in patients with asthma. Indian J Med Res. 2018;148(4):441-8.
86. Heffler E, Guida G, Marsico P, et al. Exhaled nitric oxide as a diagnostic test for asthma in rhinitic patients with asthmatic symptoms. Respir Med. 2006;100(11):1981-7.
87. Hewitt RS, Modrich CM, Medlicott T, et al. Supporting the diagnosis of non-specific respiratory symptoms in primary care: the role of exhaled nitric oxide measurement and spirometry. Prim Care Respir J. 2008;17(2):97-103.
88. Hewitt RS, Smith AD, Cowan JO, et al. Serial exhaled nitric oxide measurements in the assessment of laboratory animal allergy. J Asthma. 2008;45(2):101-7.
89. Hojo M, Shirai T, Hirashima J, et al. Comparison of the clinical effects of combined salmeterol/fluticasone delivered by dry powder or pressurized metered dose inhaler. Pulm Pharmacol Ther. 2016;37:43-8.
90. Hoshino M, Ohtawa J, Akitsu K. Effects of the addition of tiotropium on airway dimensions in symptomatic asthma. Allergy Asthma Proc. 2016;37(6):147-53.
91. Inoue H, Ito I, Niimi A, et al. Association of interleukin 1 receptor-like 1 gene polymorphisms with eosinophilic phenotype in Japanese adults with asthma. Respir Investig. 2017;55(6):338-47.

92. Inoue H, Niimi A, Matsumoto H, et al. A 12-week, randomized, parallel-group, proof-of-concept study of tulobuterol patch and salmeterol inhaler as add-on therapy in adult-onset mild-to-moderate asthma. Clinical and Experimental Pharmacology and Physiology. 2017;44(1):21-9.
93. Jaakkola JJK, Hernberg S, Lajunen TK, et al. Smoking and lung function among adults with newly onset asthma. BMJ Open Resp. 2019;6(377).
94. Jabbal S, Manoharan A, Lipworth J, et al. Is Gly16Arg beta(2) Receptor Polymorphism Related to Impulse Oscillometry in a Real-Life Asthma Clinic Setting? Lung. 2016;194(2):267-71.
95. Jacinto T, Malinovschi A, Janson C, et al. Evolution of exhaled nitric oxide levels throughout development and aging of healthy humans. J Breath Res. 2015;9(3):036005.
96. Jain VV, Abejie B, Bashir MH, et al. Lung volume abnormalities and its correlation to spirometric and demographic variables in adult asthma. J Asthma. 2013;50(6):600-5.
97. Jalota L, Allison DR, Prajapati V, et al. Ability of Exhaled Nitric Oxide to Discriminate for Airflow Obstruction Among Frequent Exacerbators of Clinically Diagnosed Asthma. Lung. 2018;196(4):455-62.
98. James AL, Knuiman MW, Divitini ML, et al. Risk factors for respiratory symptoms in adults: the Busselton Health Study. Respirology. 2013;18(8):1256-60.
99. Jo EJ, Song WJ, Kim TW, et al. Reference ranges and determinant factors for exhaled nitric oxide in a healthy Korean adult population. Allergy. 2013;68:76-.
100. Johansson EL, Ternesten-Hasseus E, Gustafsson P, et al. Small and large airway reactions to osmotic stimuli in asthma and chronic idiopathic cough. Pulm Pharmacol Ther. 2018;49:112-8.
101. Jones AD, Homan AC, Favell DJ, et al. Investigation of the levels of N(tau)-Methylhistidine in a range of beef cuts and offals. Meat Sci. 1985;15(3):137-47.
102. Kamada T, Kaneko M, Tomioka H. The relationship between respiratory system impedance and lung function in asthmatics: A prospective observational study. Respiratory Physiology & Neurobiology. 2017;239:41-5.
103. Kamada T, Kaneko M, Tomioka H. Comparison of respiratory system impedance in asthma and COPD: A prospective observational study. Respirology. 2018;23(5):478-84.
104. Kampe M, Vosough M, Malinovschi A, et al. Upper airway and skin symptoms in allergic and non-allergic asthma: Results from the Swedish GA(2)LEN study. J Asthma. 2018;55(3):275-83.
105. Kasteleyn MJ, Bonten TN, de Mutsert R, et al. Pulmonary function, exhaled nitric oxide and symptoms in asthma patients with obesity: a cross-sectional study. Respir Res. 2017;18(1):205.
106. Katsoulis K, Ganavias L, Michailopoulos P, et al. Exhaled nitric oxide as screening tool in subjects with suspected asthma without reversibility. Int Arch Allergy Immunol. 2013;162(1):58-64.
107. Kauppi P, Jarvela M, Tuomi T, et al. Systemic inflammatory responses following welding inhalation challenge test. Toxicol Rep. 2015;2:357-64.
108. Kawamatawong T, Charoenniwassakul S, Rerkpattanapipat T. The asthma and chronic obstructive pulmonary disease overlap syndrome in tertiary care setting Thailand. Asia Pac Allergy. 2017;7(4):227-33.
109. Kermode JA, Brown NJ, Hardaker KM, et al. The effect of airway remodelling on airway hyper-responsiveness in asthma. Respir Med. 2011;105(12):1798-804.
110. Khalid I, Morris ZQ, DiGiovine B. Specific conductance criteria for a positive methacholine challenge test: are the American Thoracic Society guidelines rather generous? Respir Care. 2009;54(9):1168-74.

111. Khalid I, Obeid I, DiGiovine B, et al. Predictive Value of sGaw, FEF25-75, and FEV1 for Development of Asthma after a Negative Methacholine Challenge Test. J Asthma. 2009;46(3):284-90.
112. Kivity S, Souhrada JF. A new diagnostic test to assess airway reactivity in asthmatics. Bull Eur Physiopathol Respir. 1981;17(2):243-54.
113. Kjellberg S, Houltz BK, Zetterstrom O, et al. Clinical characteristics of adult asthma associated with small airway dysfunction. Respir Med. 2016;117:92-102.
114. Klein G, Ruhle KH, Matthys H. The inhalatory propranolol provocation test - a new procedure for differentiating between healthy subjects and asthmatics. Praxis und klinik der pneumologie. 1988;42:287-92.
115. Koruga D, Baletic N, Veres KT, et al. Impulse oscillometry in evaluation bronchial hyperresponsiveness in patients with persistent allergic rhinitis. Vojnosanitetski Pregled. 2018;75(1):39-45.
116. Kostikas K, Papaioannou AI, Tanou K, et al. Portable exhaled nitric oxide as a screening tool for asthma in young adults during pollen season. Chest. 2008;133(4):906-13.
117. Kowal K, Bodzenta-Lukaszyk A, Zukowski S. Exhaled nitric oxide in evaluation of young adults with chronic cough. J Asthma. 2009;46(7):692-8.
118. Kraemer R, Smith HJ, Sigrist T, et al. Diagnostic accuracy of methacholine challenge tests assessing airway hyperreactivity in asthmatic patients - a multifunctional approach. Respir Res. 2016;17(1):154.
119. Kumar R, Gupta N. Exhaled nitric oxide atopy, and spirometry in asthma and rhinitis patients in India. Adv Respir Med. 2017;85(4):186-92.
120. Laurent F, Latrabe V, Raherison C, et al. Functional significance of air trapping detected in moderate asthma. Eur Radiol. 2000;10(9):1404-10.
121. Lee DK, Fardon TC, Bates CE, et al. Airway and systemic effects of hydrofluoroalkane formulations of high-dose ciclesonide and fluticasone in moderate persistent asthma. Chest. 2005;127(3):851-60.
122. Lee JH, Lee YW, Shin YS, et al. Exercise-induced airway obstruction in young asthmatics measured by impulse oscillometry. J Investig Allergol Clin Immunol. 2010;20(7):575-81.
123. Lehtimaki L, Kankaanranta H, Saarelainen S, et al. Increased alveolar nitric oxide concentration in asthmatic patients with nocturnal symptoms. Eur Respir J. 2002;20(4):841-5.
124. Lehtimaki L, Kankaanranta H, Saarelainen S, et al. Peripheral inflammation in patients with asthmatic symptoms but normal lung function. J Asthma. 2005;42(7):605-9.
125. Levai IK, Hull JH, Loosemore M, et al. Environmental influence on the prevalence and pattern of airway dysfunction in elite athletes. Respirology. 2016;21(8):1391-6.
126. Linna A, Oksa P, Palmroos P, et al. Respiratory health of cobalt production workers. Am J Ind Med. 2003;44(2):124-32.
127. Liu JM, Hu HC, Shi MH, et al. [The significance of volumetric capnography in assessment of asthmatic acute exacerbation staging]. Zhonghua Jie He He Hu Xi Za Zhi. 2008;31(3):186-90.
128. Liu L, Li G, Sun Y, et al. Airway wall thickness of allergic asthma caused by weed pollen or house dust mite assessed by computed tomography. Respir Med. 2015;109(3):339-46.
129. Liu L, Liu W, Liu C, et al. Study on small airway function in asthmatics with fractional exhaled nitric oxide and impulse oscillometry. Clin Respir J. 2018;12(2):483-90.
130. Liu YH, Liu T, Wu JX, et al. The Correlation between FSTL1 Expression and Airway Remodeling in Asthmatics. Mediators of Inflammation. 2017;2017.

131. Lloris Bayo A, Perpina Tordera M, Martinez Perez E, et al. [Contribution of exhaled nitric oxide measurements to abbreviated bronchial challenge test protocols]. Arch Bronconeumol. 2008;44(8):402-7.
132. Lluncor M, Barranco P, Amaya ED, et al. Relationship between upper airway diseases, exhaled nitric oxide, and bronchial hyperresponsiveness to methacholine. J Asthma. 2019;56(1):53-60.
133. Lund TK, Pedersen L, Anderson SD, et al. Are asthma-like symptoms in elite athletes associated with classical features of asthma? Br J Sports Med. 2009;43(14):1131-5.
134. Malerba M, Radaeli A, Olivini A, et al. Association of FEF25-75% Impairment with Bronchial Hyperresponsiveness and Airway Inflammation in Subjects with Asthma-Like Symptoms. Respiration. 2016;91(3):206-14.
135. Malinovschi A, Backer V, Harving H, et al. The value of exhaled nitric oxide to identify asthma in smoking patients with asthma-like symptoms. Respir Med. 2012;106(6):794-801.
136. Maneira Godinho Netto AC, dos Reis TG, Matheus CF, et al. Fraction of exhaled nitric oxide measurements in the diagnoses of asthma in elderly patients. Clin Interv Aging. 2016;11:623-9.
137. Maniscalco M, Calabrese C, D'Amato M, et al. Association between exhaled nitric oxide and nasal polyposis in severe asthma. Respir Med. 2019;152:20-4.
138. Manoharan A, Anderson WJ, Lipworth J, et al. Assessment of spirometry and impulse oscillometry in relation to asthma control. Lung. 2015;193(1):47-51.
139. Manoharan A, von Wilamowitz-Moellendorff A, Morrison A, et al. Effects of formoterol or salmeterol on impulse oscillometry in patients with persistent asthma. J Allergy Clin Immunol. 2016;137(3):727-33 e1.
140. Mariotta S, Sposato B, Ricci A, et al. Reversibility test in the early stages of bronchial asthma. J Asthma. 2005;42(6):487-91.
141. Martin MJ, Wilson E, Gerrard-Tarpey W, et al. The utility of exhaled nitric oxide in patients with suspected asthma. Thorax. 2016;71(6):562-4.
142. Mason P, Scarpa MC, Guarnieri G, et al. Exhaled nitric oxide dynamics in asthmatic reactions induced by diisocyanates. Clin Exp Allergy. 2016;46(12):1531-9.
143. Mathov E, Jares DM. Successive effects of a vagolitic and a betadrenergic in the differential diagnosis of nonimmunologic and allergic asthma. Allergol Immunopathol (Madr). 1984;12(4):293-302.
144. Matsunaga K, Hirano T, Akamatsu K, et al. Exhaled nitric oxide cutoff values for asthma diagnosis according to rhinitis and smoking status in Japanese subjects. Allergology international : official journal of the Japanese Society of Allergology. 2011;60(3):331-7.
145. Matsunaga K, Hirano T, Oka A, et al. Persistently high exhaled nitric oxide and loss of lung function in controlled asthma. Allergology international : official journal of the Japanese Society of Allergology. 2016;65(3):266-71.
146. Miedinger D, Chhajed PN, Stolz D, et al. Reliability and validity of a German asthma quality of life questionnaire. Swiss Med Wkly. 2006;136(5-6):89-95.
147. Miedinger D, Mosimann N, Meier R, et al. Asthma tests in the assessment of military conscripts. Clin Exp Allergy. 2010;40(2):224-31.
148. Mikos M, Grzanka P, Sladek K, et al. High-resolution computed tomography evaluation of peripheral airways in asthma patients: comparison of focal and diffuse air trapping. Respiration. 2009;77(4):381-8.
149. Miller ME, Levin L, Bernstein JA. Characterization of a population of monozygotic twins with asthma. J Asthma. 2005;42(5):325-30.
150. Millward D, Paul S, Brown M, et al. The diagnosis of asthma and exercise-induced bronchospasm in division I athletes. Clin J Sport Med. 2009;19(6):482-6.

151. Mirsadraee M, Forouzesh B, Rosh, et al. Accuracy of mid expiratory flow and dysanapsis parameters for evaluation of methacholine provocation test. Tanaffos. 2009;8(2):24-30.
152. Montuschi P, Santonico M, Mondino C, et al. Diagnostic performance of an electronic nose, fractional exhaled nitric oxide, and lung function testing in asthma. Chest. 2010;137(4):790-6.
153. Morris MJ, Madgwick RG, Collyer I, et al. Analysis of expiratory tidal flow patterns as a diagnostic tool in airflow obstruction. Eur Respir J. 1998;12(5):1113-7.
154. Munnik P, van der Lee I, Fijn J, et al. Comparison of eNO and histamine hyperresponsiveness in diagnosing asthma in new referrals. Respir Med. 2010;104(6):801-7.
155. Musk AW, Knuiman M, Hunter M, et al. Patterns of airway disease and the clinical diagnosis of asthma in the Busselton population. Eur Respir J. 2011;38(5):1053-9.
156. Naji N, Keung E, Kane J, et al. Comparison of changes in lung function measured by plethymography and IOS after bronchoprovocation. Respir Med. 2013;107(4):503-10.
157. Nanchev L. A forced expiration end-segment flow rate to improve diagnosis of reversible bronchial obstruction: a spirographic examination. Respiration. 1978;36(2):73-7.
158. Nayak UB, Morakhia NV, Acharya VK, et al. A study of fraction of exhaled nitric oxide levels as a diagnostic marker in patients with bronchial asthma. Indian Academy of Clinical Medicine. 2013;14(2):123-7.
159. Neelamegan R, Saka V, Tamilarasu K, et al. Clinical Utility of Fractional exhaled Nitric Oxide (FeNO) as a Biomarker to Predict Severity of Disease and Response to Inhaled Corticosteroid (ICS) in Asthma Patients. Journal of Clinical and Diagnostic Research. 2016;10(12):Fc1-Fc6.
160. Ngajilo D, Singh T, Ratshikhopha E, et al. Risk factors associated with allergic sensitization and asthma phenotypes among poultry farm workers. Am J Ind Med. 2018;61(6):515-23.
161. Nickels AS, Lim KG. Evaluation of exhaled nitric oxide's ability to predict methacholine challenge in adults with nonobstructive spirometry. Ann Allergy Asthma Immunol. 2016;117(4):365-9 e1.
162. Nilsen K, Gove K, Thien F, et al. Comparison of two methods of determining lung de-recruitment, using the forced oscillation technique. European Journal of Applied Physiology. 2018;118(10):2213-24.
163. Novkovic D, Skuletic V, Vulin A, et al. Exercise-induced bronchoconstriction and non-specific airway hyperreactivity in patients suffering from bronchial asthma. Vojnosanit Pregl. 2014;71(2):191-4.
164. Ntontsi P, Loukides S, Bakakos P, et al. Clinical, functional and inflammatory characteristics in patients with paucigranulocytic stable asthma: Comparison with different sputum phenotypes. Allergy. 2017;72(11):1761-7.
165. Ostapkovich VE, Pankova VB. [Diagnosis of pre-asthmatic conditions in workers in chemical plants]. Vestn Otorinolaringol. 1980(6):64-7.
166. Osthoff M, Michel F, Strupler M, et al. Bronchial hyperresponsiveness testing in athletes of the Swiss Paralympic team. BMC Sports Sci Med Rehabil. 2013;5(1):7.
167. Paggiaro PL, Chan Yeung M. Pattern of specific airway response in asthma due to western red cedar (Thuja plicata): relationship with length of exposure and lung function measurements. Clin Allergy. 1987;17(4):333-9.
168. Papakosta D, Latsios D, Manika K, et al. Asthma control test is correlated to FEV1 and nitric oxide in Greek asthmatic patients: influence of treatment. J Asthma. 2011;48(9):901-6.
169. Parameswaran K, Belda J, Sears MR. Use of peak flow variability and methacholine responsiveness in predicting changes from pre-test diagnosis of asthma. Eur Respir J. 1999;14(6):1358-62.

170. Park JW, Lee YW, Jung YH, et al. Impulse oscillometry for estimation of airway obstruction and bronchodilation in adults with mild obstructive asthma. Ann Allergy Asthma Immunol. 2007;98(6):546-52.
171. Pedrosa M, Cancelliere N, Barranco P, et al. Usefulness of exhaled nitric oxide for diagnosing asthma. J Asthma. 2010;47(7):817-21.
172. Pelicaric D, Petanjek BB, Jurisic MK. Relationship between the exhaled nitric oxide and airway hyperresponsiveness in patients with asthma. [German]. Atemwegs- und Lungenkrankheiten. 2008;34(7):261-5.
173. Pereira CA, Mendonca EM, Sato T, et al. [Bronchial provocation test with carbachol in the diagnosis of asthma. Report of cases and comments]. Rev Paul Med. 1984;102(4):140-4.
174. Petanjek BB, Grle SP, Vrankovic D, et al. Variability of lung function parameters in patients with persistent allergic asthma. [German]. Atemwegs- und Lungenkrankheiten. 2010;36(8):310-6.
175. Petsky HL, Cates CJ, Kew KM, et al. Tailoring asthma treatment on eosinophilic markers (exhaled nitric oxide or sputum eosinophils): a systematic review and meta-analysis. Thorax. 2018;73(12):1110-9.
176. Petsky HL, Kew KM, Turner C, et al. Exhaled nitric oxide levels to guide treatment for adults with asthma. Cochrane Database Syst Rev. 2016;9(9):CD011440.
177. Ponte EV, Souza-Machado A, Souza-Machado C, et al. Atopy is not associated with poor control of asthma. J Asthma. 2012;49(10):1021-6.
178. Porpodis K, Domvri K, Kontakiotis T, et al. Comparison of diagnostic validity of mannitol and methacholine challenges and relationship to clinical status and airway inflammation in steroid-naive asthmatic patients. J Asthma. 2017;54(5):520-9.
179. Porsbjerg C, Rasmussen L, Thomsen SF, et al. Response to mannitol in asymptomatic subjects with airway hyper-responsiveness to methacholine. Clin Exp Allergy. 2007;37(1):22-8.
180. Porsbjerg C, Sverrild A, Backer V. Combining the Mannitol Test and FeNO in the Assessment of Poorly Controlled Asthma. The journal of allergy and clinical immunology In practice. 2015;3(4):553-9.
181. Postma DS, Brightling C, Baldi S, et al. Exploring the relevance and extent of small airways dysfunction in asthma (ATLANTIS): baseline data from a prospective cohort study. Lancet Respir Med. 2019;7(5):402-16.
182. Price OJ, Ansley L, Hull JH. Diagnosing Exercise-Induced Bronchoconstriction With Eucapnic Voluntary Hyperpnea: Is One Test Enough? The journal of allergy and clinical immunology In practice. 2015;3(2):243-9.
183. Prieto L, Bruno L, Gutierrez V, et al. Airway responsiveness to adenosine 5 '-monophosphate and exhaled nitric oxide measurements - Predictive value as markers for reducing the dose of inhaled corticosteroids in asthmatic subjects. Chest. 2003;124(4):1325-33.
184. Ramirez D, Patel P, Casillas A, et al. Assessment of high-sensitivity C-reactive protein as a marker of airway inflammation in asthma. Ann Allergy Asthma Immunol. 2010;104(6):485-9.
185. Rice SG, Bierman CW, Shapiro GG, et al. Identification of exercise-induced asthma among intercollegiate athletes. Ann Allergy. 1985;55(6):790-3.
186. Riley CM, Wenzel SE, Castro M, et al. Clinical Implications of Having Reduced Mid Forced Expiratory Flow Rates (FEF25-75), Independently of FEV1, in Adult Patients with Asthma. Plos One. 2015;10(12).
187. Rodriguez Paredes A, Trujillo Trujillo MJ, olfo Cano M, et al. Spirometric evolution of asthmatic patients in an allergy clinic. Alergologia e inmunologia clinica. 2001;16(1):21-5.

188. Rolla G, Guida G, Heffler E, et al. Diagnostic classification of persistent rhinitis and its relationship to exhaled nitric oxide and asthma: a clinical study of a consecutive series of patients. Chest. 2007;131(5):1345-52.
189. Rosenkranz SK, Swain KE, Rosenkranz RR, et al. Modifiable lifestyle factors impact airway health in non-asthmatic prepubescent boys but not girls. Pediatr Pulmonol. 2011;46(5):464-72.
190. Rossall M, Cadden P, Kolsum U, et al. A comparison of the clinical and induced sputum characteristics of early- and late-onset asthma. Lung. 2012;190(4):459-62.
191. Rouhos A, Ekroos H, Karjalainen J, et al. Exhaled nitric oxide and exercise-induced bronchoconstriction in young male conscripts: association only in atopics. Allergy. 2005;60(12):1493-8.
192. Saadeh C, Cross B, Saadeh C, et al. Retrospective observations on the ability to diagnose and manage patients with asthma through the use of impulse oscillometry: comparison with spirometry and overview of the literature. Pulm Med. 2014;2014:376890.
193. Sanguinetti CM, Gasparini S, Bonifazi F, et al. Exercise-induced asthma diagnosis and prevention with a metered dose aerosol formulation of sodium cromoglycate. Respiration. 1982;43(2):132-41.
194. Sano H, Tomita K, Sano A, et al. Accuracy of objective tests for diagnosing adult asthma in symptomatic patients: A systematic literature review and hierarchical Bayesian latent-class meta-analysis. Allergology international : official journal of the Japanese Society of Allergology. 2019;68(2):191-8.
195. Santos A, Faria E, Geraldes L, et al. Parameters for monitoring severe asthma - A prospective study. [Portuguese, English]. Revista Portuguesa de Imunoalergologia. 2009;17(2):135-53.
196. Sastre J, Costa C, del Garcia Potro M, et al. Changes in exhaled nitric oxide after inhalation challenge with occupational agents. J Investig Allergol Clin Immunol. 2013;23(6):421-7.
197. Sato S, Saito J, Fukuhara A, et al. The clinical role of fractional exhaled nitric oxide in asthma control. Ann Allergy Asthma Immunol. 2017;119(6):541-7.
198. Sato S, Saito J, Sato Y, et al. Clinical usefulness of fractional exhaled nitric oxide for diagnosing prolonged cough. Respir Med. 2008;102(10):1452-9.
199. Schleich FN, As, ei R, et al. Is FE(NO50) useful diagnostic tool in suspected asthma? Int J Clin Pract. 2012;66(2):158-65.
200. Schleich FN, Zanella D, Stefanuto PH, et al. Exhaled Volatile Organic Compounds are Able to Discriminate between Neutrophilic and Eosinophilic Asthma. Am J Respir Crit Care Med. 2019;11.
201. Schmekel B, Smith HJ. The diagnostic capacity of forced oscillation and forced expiration techniques in identifying asthma by isocapnic hyperpnoea of cold air. Eur Respir J. 1997;10(10):2243-9.
202. Schneider A, Faderl B, Schwarzbach J, et al. Prognostic value of bronchial provocation and FENO measurement for asthma diagnosis--results of a delayed type of diagnostic study. Respir Med. 2014;108(1):34-40.
203. Schneider A, Linde K, Reitsma JB, et al. A novel statistical model for analyzing data of a systematic review generates optimal cutoff values for fractional exhaled nitric oxide for asthma diagnosis. J Clin Epidemiol. 2017;92:69-78.
204. Schneider A, Schwarzbach J, Faderl B, et al. FENO measurement and sputum analysis for diagnosing asthma in clinical practice. Respir Med. 2013;107(2):209-16.
205. Schneider A, Tilemann L, Schermer T, et al. Diagnosing asthma in general practice with portable exhaled nitric oxide measurement--results of a prospective diagnostic study: FENO

< or = 16 ppb better than FENO < or =12 ppb to rule out mild and moderate to severe asthma [added]. Respir Res. 2009;10(15):15.
206. Schneider A, Wagenpfeil G, Jorres RA, et al. Influence of the practice setting on diagnostic prediction rules using FENO measurement in combination with clinical signs and symptoms of asthma. BMJ Open. 2015;5(11):e009676.
207. Scott S, Currie J, Albert P, et al. Risk of misdiagnosis, health-related quality of life, and BMI in patients who are overweight with doctor-diagnosed asthma. Chest. 2012;141(3):616-24.
208. Sergeeva GR, Emelyanov AV, Korovina OV, et al. Severe asthma: Characteristics of patients in clinical practice. [Russian]. Terapevticheskii. 2015;87(12):26-31.
209. Seys SF, Feyen L, Keirsbilck S, et al. An outbreak of swimming-pool related respiratory symptoms: An elusive source of trichloramine in a municipal indoor swimming pool. Int J Hyg Environ Health. 2015;218(4):386-91.
210. Sharshar RS, Mohamed AS. The utility of impulse oscillometry in asthma: A comparison of spirometry versus impulse oscillometry system. Egyptian Journal of Chest Diseases and Tuberculosis. 2017;66(2):207-9.
211. Shi F, Qiu C, Yu J, et al. Comparison of Fractional Exhaled Nitric Oxide in Elderly Patients with Asthma-chronic Obstructive Pulmonary Disease Overlap and Other Airway Inflammatory Diseases. Iran J Allergy Asthma Immunol. 2018;17(3):232-9.
212. Shimoda T, Obase Y, Kishikawa R, et al. The fractional exhaled nitric oxide and serum high sensitivity C-reactive protein levels in cough variant asthma and typical bronchial asthma. Allergology international : official journal of the Japanese Society of Allergology. 2013;62(2):251-7.
213. Short PM, Anderson WJ, Manoharan A, et al. Usefulness of impulse oscillometry for the assessment of airway hyperresponsiveness in mild-to-moderate adult asthma. Ann Allergy Asthma Immunol. 2015;115(1):17-20.
214. Sin BA, Yildiz OA, Dursun AB, et al. Airway hyperresponsiveness: a comparative study of methacholine and exercise challenges in seasonal allergic rhinitis with or without asthma. J Asthma. 2009;46(5):486-91.
215. Siroux V, Boudier A, Dolgopoloff M, et al. Forced midexpiratory flow between 25% and 75% of forced vital capacity is associated with long-term persistence of asthma and poor asthma outcomes. J Allergy Clin Immunol. 2016;137(6):1709-+.
216. Smith AD, Cowan JO, Filsell S, et al. Diagnosing asthma - Comparisons between exhaled nitric oxide measurements and conventional tests. American Journal of Respiratory and Critical Care Medicine. 2004;169(4):473-8.
217. Sood N, Turcotte SE, Wasilewski NV, et al. Small-airway obstruction, dynamic hyperinflation, and gas trapping despite normal airway sensitivity to methacholine in adults with chronic cough. Journal of applied physiology (Bethesda, Md : 1985). 2019;126(2):294-304.
218. Sposato B, Mariotta S, Ricci A. When should a reversibility test be performed on patients with early stages of asthma and normal spirometry? J Asthma. 2008;45(6):479-83.
219. Stenberg H, Diamant Z, Ankerst J, et al. Small airway involvement in the late allergic response in asthma. Clin Exp Allergy. 2017;47(12):1555-65.
220. Stone B, Davis JR, Trudo F, et al. Characterizing patients with asthma who received Global Initiative for Asthma steps 4-5 therapy and managed in a specialty care setting. Allergy Asthma Proc. 2018;39(1):27-35.
221. Suzuki Y, Wakahara K, Nishio T, et al. Airway basophils are increased and activated in eosinophilic asthma. Allergy. 2017;72(10):1532-9.
222. Svenningsen S, Nair P, Guo F, et al. Is ventilation heterogeneity related to asthma control? Eur Respir J. 2016;48(2):370-9.

223. Sverrild A, Porsbjerg C, Thomsen SF, et al. Diagnostic properties of inhaled mannitol in the diagnosis of asthma: a population study. J Allergy Clin Immunol. 2009;124(5):928-32 e1.
224. Sverrild A, Porsbjerg C, Thomsen SF, et al. Airway hyperresponsiveness to mannitol and methacholine and exhaled nitric oxide: a random-sample population study. J Allergy Clin Immunol. 2010;126(5):952-8.
225. Swierczynska-Machura D, Krakowiak A, Wiszniewska M, et al. Exhaled nitric oxide levels after specific inahalatory challenge test in subjects with diagnosed occupational asthma. Int J Occup Med Environ Health. 2008;21(3):219-25.
226. Thomas M, McKinley RK, Mellor S, et al. Breathing exercises for asthma: a randomised controlled trial. Thorax. 2009;64(1):55-61.
227. Thomson NC, Chaudhuri R, Spears M, et al. Poor Symptom Control Is Associated With Reduced CT Scan Segmental Airway Lumen Area in Smokers With Asthma. Chest. 2015;147(3):735-44.
228. Thorat YT, Salvi SS, Kodgule RR. Peak flow meter with a questionnaire and mini-spirometer to help detect asthma and COPD in real-life clinical practice: a cross-sectional study. NPJ Prim Care Respir Med. 2017;27(1):32.
229. Tilemann L, Gindner L, Meyer F, et al. Differences in local and systemic inflammatory markers in patients with obstructive airways disease. Prim Care Respir J. 2011;20(4):407-14.
230. Tomari S, Matsuse H, Machida I, et al. Three 20-minute interspaced salbutamol inhalations as a test for the diagnosis of reversible airflow limitation in adult asthmatics. J Asthma. 2004;41(1):43-8.
231. Tomasiak-Lozowska MM, Misztal T, Rusak T, et al. Asthma is associated with reduced fibrinolytic activity, abnormal clot architecture, and decreased clot retraction rate. Allergy. 2017;72(2):314-9.
232. Topalovic M, Derom E, Osadnik CR, et al. Airways resistance and specific conductance for the diagnosis of obstructive airways diseases. Respir Res. 2015;16(88):88.
233. Trinkmann F, Gotzmann J, Saur D, et al. Multiple breath washout testing in adults with pulmonary disease and healthy controls - can fewer measurements eventually be more? BMC Pulm Med. 2017;17.
234. Tsai JJ, Shih JT, Lee HL, et al. Bronchoprovocation test in the normal and in asthmatics. Zhonghua Min Guo Wei Sheng Wu Ji Mian Yi Xue Za Zhi. 1986;19(2):118-23.
235. Tsilogianni Z, Hillas G, Bakakos P, et al. Sputum interleukin-13 as a biomarker for the evaluation of asthma control. Clin Exp Allergy. 2016;46(7):923-31.
236. Tsolakis N, Malinovschi A, Nordvall L, et al. The absence of serum IgE antibodies indicates non-type 2 disease in young asthmatics. Clin Exp Allergy. 2018;48(6):722-30.
237. Tsuburai T, Suzuki S, Tsurikisawa N, et al. [Use of forced oscillation technique to detect airflow limitations in adult Japanese asthmatics]. Arerugi. 2012;61(2):184-93.
238. Tsuburai T, Tsurikisawa N, Taniguchi M, et al. The relationship between exhaled nitric oxide measured with an off-line method and airway reversible obstruction in Japanese adults with asthma. Allergology international : official journal of the Japanese Society of Allergology. 2007;56(1):37-43.
239. Tworek D, Bochenska-Marciniak M, Kupczyk M, et al. [Lack of correlation between exhaled nitric oxide (eNO) and clinical indicators of the disease activity and quality of life in mild and moderate asthmatics]. Pneumonol Alergol Pol. 2006;74(4):391-5.
240. Udesen PB, Westergaard CG, Porsbjerg C, et al. Stability of FeNO and airway hyperresponsiveness to mannitol in untreated asthmatics. J Asthma. 2017;54(5):530-6.
241. Ura M, Tanaka H, Takahashi K, et al. [Value of Fractional Exhaled Nitric Oxide after Using a Beta-2 Bronchodilator in the Differential Diagnosis of Bronchial Asthma and Chronic Obstructive Pulmonary Disease]. Rinsho Byori. 2016;64(2):127-32.

242. Usmani OS, Singh D, Spinola M, et al. The prevalence of small airways disease in adult asthma: A systematic literature review. Respir Med. 2016;116:19-27.
243. Vakali S, Vogiatzis I, Florou A, et al. Exercice-induced bronchoconstriction among athletes: Assessment of bronchial provocation tests. Respiratory Physiology & Neurobiology. 2017;235:34-9.
244. van Asch CJ, Balemans WA, Rovers MM, et al. Atopic disease and exhaled nitric oxide in an unselected population of young adults. Ann Allergy Asthma Immunol. 2008;100(1):59-65.
245. Voutilainen M, Malmberg LP, Vasankari T, et al. Exhaled nitric oxide indicates poorly athlete's asthma. Clin Respir J. 2013;7(4):347-53.
246. Vukoja M, Rebic P, Lazic Z, et al. Early detection of asthma and chronic obstructive pulmonary disease in primary care patients. Med Pregl. 2013;66(1-2):46-52.
247. Wang Y, Chen P, Dai AN, et al. Intervention Studies of Inhaled Corticosteroids Combined with Long-acting Theophylline or Long-acting beta(2)-agonists in Patients with Moderate to Severe Asthma: A Randomized, Controlled Study. Clinical Therapeutics. 2016;38(12):2622-7.
248. Weatherall M, Travers J, Shirtcliffe PM, et al. Distinct clinical phenotypes of airways disease defined by cluster analysis. Eur Respir J. 2009;34(4):812-8.
249. Wei J, Ma L, Wang J, et al. Airway reversibility in asthma and phenotypes of Th2-biomarkers, lung function and disease control. Allergy Asthma Clin Immunol. 2018;14:89.
250. White EC, de Klerk N, Hantos Z, et al. Mannitol challenge testing for asthma in a community cohort of young adults. Respirology. 2017;22(4):678-83.
251. Williamson PA, Vaidyanathan S, Clearie K, et al. Airway dysfunction in nasal polyposis: a spectrum of asthmatic disease? Clin Exp Allergy. 2011;41(10):1379-85.
252. Wolfromm R, Pouillard J, Kaufman E. [Ventilatory tests in the diagnosis of asthma in adults]. Maroc Med. 1966;45(496):706-8.
253. Wu D, Li L, Zhang M, et al. Two inflammatory phenotypes of nasal polyps and comorbid asthma. Ann Allergy Asthma Immunol. 2017;118(3):318-25.
254. Yang SY, Kim YH, Byun MK, et al. Repeated measurement of fractional exhaled nitric oxide is not essential for asthma screening. J Investig Allergol Clin Immunol. 2018;28(2):98-105.
255. Yoshikawa T, Kanazawa H. Characteristics of young atopic adults with self-reported past wheeze and airway hyperresponsiveness. Allergology international : official journal of the Japanese Society of Allergology. 2012;61(1):65-73.
256. Yuan Y, Luo Y, He T, et al. [Value of measuring resistance of airway for diagnosis of asthma]. Hua Xi Yi Ke Da Xue Xue Bao. 1996;27(3):302-5.
257. Yun KH, Chi RC, Kyung HP, et al. Clinical significance of methacholine bronchial challenge test in differentiating asthma from COPD. [Korean]. Tuberculosis and Respiratory Diseases. 2006;61(5):433-9.
258. Zapletal A, Chalupova J, Svobodova T. FEV1 - A not very sensitive parameter for the assessment of induced bronchoconstriction and bronchodilation. [Czech]. Studia Pneumologica et Phthiseologica. 2005;65(1):32-9.
259. Zhang YM, Lin JT. [The values of fractional exhaled nitric oxide in the diagnosis and treatment of chronic cough]. Zhonghua Jie He He Hu Xi Za Zhi. 2011;34(7):504-8.
260. Zhao H, Li R, Lv Y, et al. Albuterol inhalation increases FeNO level in steroid-naive asthmatics but not COPD patients with reversibility. Clin Respir J. 2015;11(3):328-36.
261. Zhu Z, Xie Y, Guan W, et al. FeNO for detecting lower airway involvement in patients with allergic rhinitis. Exp Ther Med. 2016;12(4):2336-40.
262. Zietkowski Z, Bodzenta-Lukaszyk A, Tomasiak MM, et al. The role of measurement of exhaled nitric oxide in asthma patients. [Polish]. Pol Arch Med Wewn. 2005;113(1):35-41.

263. Zietkowski Z, Bodzenta-Lukaszyk A, Tomasiak MM, et al. Comparison of exhaled nitric oxide measurement with conventional tests in steroid-naive asthma patients. J Investig Allergol Clin Immunol. 2006;16(4):239-46.
264. Zietkowski Z, Tomasiak MM, Skiepko R, et al. RANTES in exhaled breath condensate of stable and unstable asthma patients. Respir Med. 2008;102(8):1198-202.
265. Baarnes CB, Thuesen BH, Linneberg A, et al. Determinants of airflow limitation in Danish adults - findings from the Health2006 cohort. Int J Chron Obstruct Pulmon Dis. 2019;14:713-8.
266. Baptist AP, Sengupta R, Pranathiageswaran S, et al. Evaluation of exhaled nitric oxide measurements in the emergency department for patients with acute asthma. Ann Allergy Asthma Immunol. 2008;100(5):415-9.
267. Berger KI, Kalish S, Shao YZ, et al. Isolated Small Airway Reactivity During Bronchoprovocation as a Mechanism for Respiratory Symptoms in WTC Dust-Exposed Community Members. American Journal of Industrial Medicine. 2016;59(9):767-76.
268. Buslau A, Voss S, Herrmann E, et al. Can we predict allergen-induced asthma in patients with allergic rhinitis? Clin Exp Allergy. 2014;44(12):1494-502.
269. Cipr, i G, Signori A, et al. Relationship between bronchial hyperreactivity and bronchodilation in patients with allergic rhinitis. Ann Allergy Asthma Immunol. 2011;106(6):460-6.
270. Cirillo I, Gallo F, Ciprandi G. Could routine spirometry suggest sensitisation in the military medicine setting? J R Army Med Corps. 2018;164(1):58-60.
271. Dressler M, Salzmann-Manrique E, Zielen S, et al. Exhaled NO as a predictor of exercise-induced asthma in cold air. Nitric Oxide-Biol Ch. 2018;76:45-52.
272. Drks. Assessment of small airway diseases by inert gas washout testing. Evaluation of N2-single and multiple breath washout versus a double tracer single breath technique in healthy adults and patients with small airway diseases 2012. Available from: https://www.cochranelibrary.com/central/doi/10.1002/central/CN-01832061/full.
273. Ekstrand Y, Ternesten-Hasseus E, Arvidsson M, et al. Sensitivity to environmental irritants and capsaicin cough reaction in patients with a positive methacholine provocation test before and after treatment with inhaled corticosteroids. J Asthma. 2011;48(5):482-9.
274. Franklin PJ, Stick SM, Le Souef PN, et al. Measuring exhaled nitric oxide levels in adults: the importance of atopy and airway responsiveness. Chest. 2004;126(5):1540-5.
275. Ghani N, Tariq F, Hassan S. Respiratory and physical ailments correlated with occupational exposure among welders in Pakistan. Journal of the Pakistan Medical Association. 2017;67(12):1910-3.
276. Gilbert R, Auchincloss JH, Jr. The interpretation of the spirogram. How accurate is it for 'obstruction'? Arch Intern Med. 1985;145(9):1635-9.
277. Ibeneme S, Egbosionu V, Ibeneme G, et al. Evidence of Allergic Reactions and Cardiopulmonary Impairments among Traders Operating from Foodstuff Warehouses. Biomed Research International. 2016;2016.
278. Kjeldgaard P, Lykkegaard J, Spillemose H, et al. Multicenter study of the COPD-6 screening device: feasible for early detection of chronic obstructive pulmonary disease in primary care? Int J Chron Obstruct Pulmon Dis. 2017;12:2323-31.
279. Krantz C, Janson C, Hollsing A, et al. Exhaled and nasal nitric oxide in relation to lung function, blood cell counts and disease characteristics in cystic fibrosis. J Breath Res. 2017;11(2):026001.
280. Lai KF, Lin L, Liu BJ, et al. Eosinophilic airway inflammation is common in subacute cough following acute upper respiratory tract infection. Respirology. 2016;21(4):683-8.
281. Linkosalo L, Lehtimaki L, Laitinen J, et al. Increased bronchial NO output in severe atopic eczema in children and adolescents. Pediatr Allergy Immunol. 2008;19(5):426-32.

282. Loymans RJB, Honkoop PJ, Termeer EH, et al. Identifying patients at risk for severe exacerbations of asthma: development and external validation of a multivariable prediction model. Thorax. 2016;71(9):838-46.
283. Malerba M, Damiani G, Carpagnano GE, et al. Values in Elderly People for Exhaled Nitric Oxide Study. Rejuvenation Res. 2016;19(3):233-8.
284. Malinovschi A, Janson C, Borres M, et al. Simultaneously increased fraction of exhaled nitric oxide levels and blood eosinophil counts relate to increased asthma morbidity. J Allergy Clin Immunol. 2016;138(5):1301-8 e2.
285. Medelli J, Lounana J, Messan F, et al. Testing of pulmonary function in a professional cycling team. J Sports Med Phys Fitness. 2006;46(2):298-306.
286. Milota T, Bloomfield M, Parackova Z, et al. Bronchial Asthma and Bronchial Hyperresponsiveness and Their Characteristics in Patients with Common Variable Immunodeficiency. International Archives of Allergy and Immunology. 2019;178(2):192-200.
287. Murri V, Antoniazzi F, Piazza M, et al. Lung Function in Women with Idiopathic Central Precocious Puberty: A Pilot Study. Horm Res Paediatr. 2017;87(2):95-102.
288. Ohrmalm L, Malinovschi A, Wong M, et al. Presence of rhinovirus in the respiratory tract of adolescents and young adults with asthma without symptoms of infection. Respir Med. 2016;115:1-6.
289. Pohjantahti H, Laitinen J, Parkkari J. Exercise-induced bronchospasm among healthy elite cross country skiers and non-athletic students. Scand J Med Sci Sports. 2005;15(5):324-8.
290. Price OJ, Ansley L, Bikov A, et al. The role of impulse oscillometry in detecting airway dysfunction in athletes. J Asthma. 2016;53(1):62-8.
291. Rentzhog CH, Janson C, Berglund L, et al. Overall and peripheral lung function assessment by spirometry and forced oscillation technique in relation to asthma diagnosis and control. Clinical and Experimental Allergy. 2017;47(12):1546-54.
292. Rex CE, Eckerstrom F, Heiberg J, et al. Surgical closure of a ventricular septal defect in early childhood leads to altered pulmonary function in adulthood: A long-term follow-up. Int J Cardiol. 2019;274:100-5.
293. Ross RG. The prevalence of reversible airway obstruction in professional football players. Med Sci Sports Exerc. 2000;32(12):1985-9.
294. Rundell KW, Im J, Mayers LB, et al. Self-reported symptoms and exercise-induced asthma in the elite athlete. Med Sci Sports Exerc. 2001;33(2):208-13.
295. Tagiyeva N, Teo E, Fielding S, et al. Occupational exposure to asthmagens and adult onset wheeze and lung function in people who did not have childhood wheeze: A 50-year cohort study. Environ Int. 2016;94:60-8.
296. Tang S, Lai P, Lai M, et al. Topical anesthesia in transconjunctival sutureless 25-gauge vitrectomy for macular-based disorders. Ophthalmologica. 2007;221(1):65-8.
297. Tantilipikorn P, Juntabenjapat J, Thongngarm T, et al. Prevalence of impaired lower airway function in Thai patients with allergic rhinitis. Asian Biomed. 2016;10(1):67-74.
298. Ulrik CS, Svenningsen C. High prevalence of asthma in Danish elite canoe- and kayak athletes. Dan Med J. 2012;59(4):A4405.
299. Van der Walt A, Baatjies R, Singh T, et al. Environmental factors associated with baseline and serial changes in fractional exhaled nitric oxide (FeNO) in spice mill workers. Occup Environ Med. 2016;73(9):614-20.
300. Vedal S, Chan-Yeung M, Enarson D, et al. Symptoms and pulmonary function in western red cedar workers related to duration of employment and dust exposure. Arch Environ Health. 1986;41(3):179-83.

301. Wang W, Xian M, Xie Y, et al. Aggravation of airway inflammation and hyper-responsiveness following nasal challenge with Dermatophagoides pteronyssinus in perennial allergic rhinitis without symptoms of asthma. Allergy. 2016;71(3):378-86.
302. West AJ, Burton D, Bell A. The association of body mass index with airway obstruction in non-asthmatics: implications for the inaccurate differential diagnosis of asthma in obesity. Canadian Journal of Respiratory Therapy. 2011;47(2):11-22.
303. Zebrowska A, Gluchowska B, Jastrzebski D, et al. Endurance Training and the Risk of Bronchial Asthma in Female Cross-Country Skiers2015. 29-34 p.
304. Zuskin E, Kanceljak B, Schachter EN, et al. Respiratory function and immunological status in cocoa and flour processing workers. Am J Ind Med. 1998;33(1):24-32.
305. Anderson WJ, Lipworth BJ. Relationship of mannitol challenge to methacholine challenge and inflammatory markers in persistent asthmatics receiving inhaled corticosteroids. Lung. 2012;190(5):513-21.
306. Asano T, Takemura M, Kanemitsu Y, et al. Combined measurements of fractional exhaled nitric oxide and nasal nitric oxide levels for assessing upper airway diseases in asthmatic patients. J Asthma. 2018;55(3):300-9.
307. Bardsley G, Daley-Yates P, Baines A, et al. Anti-inflammatory duration of action of fluticasone furoate/vilanterol trifenatate in asthma: a cross-over randomised controlled trial. Respir Res. 2018;19(1):133.
308. Baumann JM, Rundell KW, Evans TM, et al. Effects of cysteine donor supplementation on exercise-induced bronchoconstriction. Med Sci Sports Exerc. 2005;37(9):1468-73.
309. Behndig AF, Larsson N, Brown JL, et al. Proinflammatory doses of diesel exhaust in healthy subjects fail to elicit equivalent or augmented airway inflammation in subjects with asthma. Thorax. 2011;66(1):12-9.
310. Bellier M, Barnig C, Renaudin JM, et al. Importance of specific inhalation challenge in the diagnosis of occupational asthma induced by quaternary ammonium compounds. The journal of allergy and clinical immunology In practice. 2015;3(5):819-20.
311. Bilgin G, Arslan H, Balci N, et al. Acupuncture for the Treatment of Mild or Moderate Asthma: A Randomized, Placebo-Controlled Clinical Trial. Nobel Medicus. 2016;12(2):31-7.
312. Caminati M, Caimmi C, Dama A, et al. What lies beyond Asthma Control Test: Suggestions for clinical practice. J Asthma. 2016;53(6):559-62.
313. Colak Y, Afzal S, Nordestgaard BG, et al. Combined value of exhaled nitric oxide and blood eosinophils in chronic airway disease: the Copenhagen General Population Study. Eur Respir J. 2018;52(2).
314. Crespo Lessmann A, Giner J, Torrego A, et al. Usefulness of the Exhaled Breath Temperature Plateau in Asthma Patients. Respiration. 2015;90(2):111-7.
315. Diaz-Guzman E, Khosravi M, Mannino DM. Asthma, chronic obstructive pulmonary disease, and mortality in the U.S. population. Copd. 2011;8(6):400-7.
316. Dixon AE, Subramanian M, DeSarno M, et al. A pilot randomized controlled trial of pioglitazone for the treatment of poorly controlled asthma in obesity. Respir Res. 2015;16:143.
317. Dressel H, Gross C, de la Motte D, et al. Educational intervention decreases exhaled nitric oxide in farmers with occupational asthma. Eur Respir J. 2007;30(3):545-8.
318. D'Souza W, Lewis S, Cheng S, et al. The prevalence of asthma symptoms, bronchial hyperresponsiveness and atopy in New Zealand adults. N Z Med J. 1999;112(1089):198-202.
319. Fujimura M, Ohkura N, Abo M, et al. Exhaled nitric oxide levels in patients with atopic cough and cough variant asthma. Respirology. 2008;13(3):359-64.
320. Gelb AF, Yamamoto A, Verbeken EK, et al. Further Studies of Unsuspected Emphysema in Nonsmoking Patients With Asthma With Persistent Expiratory Airflow Obstruction. Chest. 2018;153(3):618-29.

321. Hashimoto S, Rijssenbeek-Nouwens LH, Fieten KB, et al. Predictors of benefit from high-altitude climate therapy in adults with severe asthma. Neth J Med. 2018;76(5):218-25.
322. Hurwitz KM, Argyros GJ, Roach JM, et al. Interpretation of eucapnic voluntary hyperventilation in the diagnosis of asthma. Chest. 1995;108(5):1240-5.
323. Jerschow E, Ren Z, Hudes G, et al. Utility of low-dose oral aspirin challenges for diagnosis of aspirin-exacerbated respiratory disease. Ann Allergy Asthma Immunol. 2016;116(4):321-8 e1.
324. Kazaks AG, Uriu-Adams JY, Albertson TE, et al. Effect of oral magnesium supplementation on measures of airway resistance and subjective assessment of asthma control and quality of life in men and women with mild to moderate asthma: a randomized placebo controlled trial. J Asthma. 2010;47(1):83-92.
325. Kirsten AM, Watz H, Brindicci C, et al. Effects of beclomethason/formoterol and budesonide/formoterol fixed combinations on lung function and airway inflammation in patients with mild to moderate asthma--an exploratory study. Pulm Pharmacol Ther. 2015;31:79-84.
326. Kjellberg S, Viklund E, Robinson PD, et al. Utility of single versus multiple breath washout in adult asthma. Clin Physiol Funct Imaging. 2018;38(6):936-43.
327. Ko FWS, Leung TF, Hui DSC, et al. Asthma Control Test correlates well with the treatment decisions made by asthma specialists. Respirology. 2009;14(4):559-66.
328. Latorre M, Baldini C, Seccia V, et al. Asthma Control and Airway Inflammation in Patients with Eosinophilic Granulomatosis with Polyangiitis. The journal of allergy and clinical immunology In practice. 2016;4(3):512-9.
329. Louhelainen N, Rytila P, Obase Y, et al. The value of sputum 8-isoprostane in detecting oxidative stress in mild asthma. J Asthma. 2008;45(2):149-54.
330. Malagrino L, Catapano G, Novelli F, et al. Markers of small airway involvement and asthma control in patients with moderate-to-severe asthma. Ann Allergy Asthma Immunol. 2014;112(6):551-2.
331. Mannix ET, Roberts M, Fagin DP, et al. The prevalence of airways hyperresponsiveness in members of an exercise training facility. J Asthma. 2003;40(4):349-55.
332. Mansournia MA, Jamali M, Mansournia N, et al. Exercise-induced bronchospasm among students of Tehran University of Medical Sciences in 2004. Allergy Asthma Proc. 2007;28(3):348-52.
333. Marsden PA, Satia I, Ibrahim B, et al. Objective Cough Frequency, Airway Inflammation, and Disease Control in Asthma. Chest. 2016;149(6):1460-6.
334. Mehrparvar AH, Hossein Davari M, Salmani Nodooshan M, et al. Assessment of bronchodilator response in various spirometric patterns. Tanaffos. 2013;12(2):28-33.
335. Michils A, Haccuria A, Michiels S, et al. Airway calibre variation is a major determinant of exhaled nitric oxide's ability to capture asthma control. Eur Respir J. 2017;50(2).
336. Motomura C, Odajima H, Tezuka J, et al. Effect of Age on Relationship Between Exhaled Nitric Oxide and Airway Hyperresponsiveness in Asthmatic Children. Chest. 2009;136(2):519-25.
337. Munoz-Lopez F, Rios-Alcolea M. The interest of FEF(25-75) in evaluating bronchial hyperresponsiveness with the methacholine test. Allergol Immunopathol (Madr). 2012;40(6):352-6.
338. Nittner-Marszalska M, Dor-Wojnarowska A, Wolanczyk-Medrala A, et al. Studying allergic inflammation and spirometry over menstrual cycles in well-controlled asthmatic women: Changes in progesterone and estradiol affect neither FENO levels nor lung function. Nitric Oxide-Biol Ch. 2018;75:95-100.

339. Pepper AN, Bulkhi A, Smith CR, et al. Effects of Exposure to New Car Interiors in Patients With Asthma and Allergic Rhinitis. Allergy Rhinol (Providence). 2018;9(2):2152656718800060.
340. Petsky HL, Cates CJ, Li A, et al. Tailored interventions based on exhaled nitric oxide versus clinical symptoms for asthma in children and adults. Cochrane Database Syst Rev. 2009(4):CD006340.
341. Powell H, Murphy VE, Taylor DR, et al. Management of asthma in pregnancy guided by measurement of fraction of exhaled nitric oxide: a double-blind, randomised controlled trial. Lancet. 2011;378(9795):983-90.
342. Razi E, Ehteram H, Akbari H, et al. Evaluation of high-sensitivity C-reactive protein in acute asthma. Tanaffos. 2012;11(1):32-7.
343. Ricciardolo FL, Sorbello V, Bellezza Fontana R, et al. Exhaled nitric oxide in relation to asthma control: A real-life survey. Allergol Immunopathol (Madr). 2016;44(3):197-205.
344. Ritz T, Kullowatz A, Bill MN, et al. Daily life negative mood and exhaled nitric oxide in asthma. Biol Psychol. 2016;118:176-83.
345. Ritz T, Rosenfield D, Steele AM, et al. Controlling asthma by training of Capnometry-Assisted Hypoventilation (CATCH) vs slow breathing: a randomized controlled trial. Chest. 2014;146(5):1237-47.
346. Ross JA, Yang Y, Song PX, et al. Quality of life, health care utilization, and control in older adults with asthma. The journal of allergy and clinical immunology In practice. 2013;1(2):157-62.
347. Rouhos A, Ekroos H, Karjalainen J, et al. Smoking attenuates increase in exhaled nitric oxide in atopic but not in nonatopic young adults with asthma. Int Arch Allergy Immunol. 2010;152(3):226-32.
348. Prieto L, Ruiz-Jimenez L, Marin J. The effect of spirometry on bronchial and alveolar nitric oxide in subjects with asthma. J Asthma. 2012;185.
349. Sekiya K, Taniguchi M, Fukutomi Y, et al. Actual control state of intermittent asthma classified on the basis of subjective symptoms. Internal medicine (Tokyo, Japan). 2011;50(15):1545-51.
350. Selge C, Thomas S, Nowak D, et al. Asthma prevalence in German Olympic athletes: A comparison of winter and summer sport disciplines. Respir Med. 2016;118:15-21.
351. Senna G, Passalacqua G, Schiappoli M, et al. Correlation among FEV, nitric oxide and asthma control test in newly diagnosed asthma. Allergy. 2007;62(2):207-8.
352. Sharifi A, Ansarin K. Effect of gastroesophageal reflux disease on disease severity and characteristics of lung functional changes in patients with asthma. J Cardiovasc Thorac Res. 2014;6(4):223-8.
353. Shimoda T, Obase Y, Kishikawa R, et al. Assessment of anti-inflammatory effect from addition of a long-acting beta-2 agonist to inhaled corticosteroid. Allergy Asthma Proc. 2016;37(5):387-93.
354. Short PM, Williamson PA, Lipworth BJ. Sensitivity of impulse oscillometry and spirometry in beta-blocker induced bronchoconstriction and beta-agonist bronchodilatation in asthma. Ann Allergy Asthma Immunol. 2012;109(6):412-5.
355. Silkoff PE, Strambu I, Laviolette M, et al. Asthma characteristics and biomarkers from the Airways Disease Endotyping for Personalized Therapeutics (ADEPT) longitudinal profiling study. Respir Res. 2015;16:142.
356. Smith AD, Cowan JO, Brassett KP, et al. Use of exhaled nitric oxide measurements to guide treatment in chronic asthma. New England Journal of Medicine. 2005;352(21):2163-73.
357. Smith AD, Cowan JO, Taylor DR. Exhaled nitric oxide levels in asthma: Personal best versus reference values. J Allergy Clin Immunol. 2009;124(4):714-8 e4.

358. Sue-Chu M, Larsson L, Bjermer L. Prevalence of asthma in young cross-country skiers in central Scandinavia: Differences between Norway and Sweden. Respir Med. 1996;90(2):99-105.
359. Svenningsen S, Kirby M, Starr D, et al. What are ventilation defects in asthma? Thorax. 2014;69(1):63-71.
360. Sverrild A, Malinovschi A, Porsbjerg C, et al. Predicting airway hyperreactivity to mannitol using exhaled nitric oxide in an unselected sample of adolescents and young adults. Respir Med. 2013;107(1):150-2.
361. Tajiri T, Niimi A, Matsumoto H, et al. Comprehensive efficacy of omalizumab for severe refractory asthma: a time-series observational study. Ann Allergy Asthma Immunol. 2014;113(4):470-5 e2.
362. Tang W, Zhou J, Miao L, et al. Clinical features in patients of cough variant asthma with normal and high level of exhaled fractional nitric oxide. Clin Respir J. 2018;12(2):595-600.
363. Wang Y, Li L, Han R, et al. Diagnostic value and influencing factors of fractional exhaled nitric oxide in suspected asthma patients. Int J Clin Exp Pathol. 2015;8(5):5570-6.
364. Wilson AM, Dempsey OJ, Sims EJ, et al. Subjective and objective markers of treatment response in patients with seasonal allergic rhinitis. Ann Allergy Asthma Immunol. 2000;85(2):111-4.
365. Woodcock A, Vestbo J, Bakerly ND, et al. Effectiveness of fluticasone furoate plus vilanterol on asthma control in clinical practice: an open-label, parallel group, randomised controlled trial. Lancet. 2017;390(10109):2247-55.
366. Yamamoto S, Miyoshi S, Katayama H, et al. Use of the forced-oscillation technique to estimate spirometry values. Int J Chron Obstruct Pulmon Dis. 2017;12:2859-68.
367. Yokoyama T. Diagnostic significance of arterial-alveolar nitrogen tension difference in assessment of ventilation-perfusion ratio inequality. Chest. 1972;62(2):191-8.
368. Zhang L, Gang J, Zhigang C, et al. Irreversible airway obstruction assessed by high-resolution computed tomography (HRCT), exhaled nitric oxide (FENO), and biological markers in induced sputum in patients with asthma. Wien Klin Wochenschr. 2014;126(17-18):515-23.
369. Amelink M, de Nijs S, de Groot C, et al. Nasal Polyposis Identifies An At Risk Phenotype Among Patients With Adult-Onset Asthma. American Journal of Respiratory and Critical Care Medicine. 2011;183(1).
370. Amirneni A, Roychowdhury P, Badwal J, et al. Use of Spirometry for Diagnosis and Management of Asthma in a Community Health Center. Ann Allergy Asthma Immunol. 2018;121(5):S9-S.
371. Anonymous. Airway hyperresponsiveness and airway inflammation in elite swimmers. Clin Respir J. 2009;3(1):62.
372. Arron JR, Holweg CT, Eng C, et al. Range And Distribution Of Type 2 Inflammatory Biomarkers In Children And Adolescents With And Without Asthma. American Journal of Respiratory and Critical Care Medicine. 2014;189.
373. Baccioglu A, Kalpaklioglu AF, Oyman TG. Occupational respiratory diseases in mosque workers. Allergy. 2017;72:636-7.
374. Backer V, Sverrild A, Ulrik CS, et al. Diagnosing asthma in a real life setting-which test to use? American Journal of Respiratory and Critical Care Medicine. 2014;189.
375. Badyda A, Chcialowski A, Dabrowiecki P, et al. Coal and wood burning products as a risk factor of respiratory and cardiovascular diseases - preliminary results of household PM2.5 emissions on health risk. Eur Respir J. 2017;50.
376. Basa Akdogan B, Koca Kalkan I, Koycu Buhari G, et al. What is the best way to diagnose asthma in patients without reversibility? Allergy: European Journal of Allergy and Clinical Immunology. 2018;73:523.

377. Berti A, Licini A, Lombardi C, et al. Small airway dysfunction in asthma: A real life study. Eur Respir J. 2018;52.
378. Berti A, Licini A, Lombardi C, et al. Small airway dysfunction in elderly patient with asthma: a real life study. Eur Respir J. 2018;52.
379. Brigham E, Boyce D, McCormack M. Variable Extrathoracic Obstruction Correlates With Higher Body Mass Index Among Adults With Asthma. Chest. 2013;144(4).
380. Burnett M, Wegienka G, Havstad S, et al. The Relationship of Fractional Exhaled Nitric Oxide Levels to Allergy and Asthma Biomarkers in Young Adults. J Allergy Clin Immunol. 2011;127(2):Ab58-Ab.
381. Carneiro-Leao L, Martins C, Vilela A, et al. Overweight effects in lung function and dyspnoea perception during methacoline challenge test. Allergy. 2016;71:531-.
382. Chai JJ, Cai BQ. The Normal Value Measurement of Fractional Concentration of Exhaled Nitric Oxide in Chinese Adults. Respirology. 2011;16:196-7.
383. Chen FJ, Huang XY, Lin GP, et al. Validity of FENO and small airway function indices in diagnosis of CVA. Eur Respir J. 2017;50.
384. Cottee A, Seccombe L, Thamrin C, et al. Bronchodilator Response in Asthma Using the Forced Oscillation Technique Is Comparable to Spirometry and Relates to Asthma Control. Respirology. 2019;24:74-.
385. Dales R, Cakmak S. Is residential ambient air limonene associated with asthma in the Canadian population? Eur Respir J. 2017;50.
386. De Vries R, Dagelet JWF, Frey U, et al. Assessment of repeatability of eNose (SpiroNose) measurements in healthy and asthmatic subjects. Eur Respir J. 2018;52.
387. Dey D, Paul M, Sengupta S, et al. Does FEF25-75 distinguish between airway obstruction and restriction in DPLD? Respirology. 2018;23:78-9.
388. Dey D, Saha D, Paul M, et al. Fef25-75 Is the Better Diagnostic Tool for Identifying Asthma Patients. Respirology. 2018;23:90-.
389. Dilka E, Tashi E, Nushi E, et al. The use of FENO in COPD: the relationship to pulmonary function tests and its importance in differential diagnosis. Eur Respir J. 2017;50.
390. Dummer J, Cowan J, Tewhaiti-Smith J, et al. Lung Health in New Zealand Gang Members: Results from a Health Hui. Respirology. 2018;23:208-.
391. Fingleton J, Williams M, Travers J, et al. Prevalence of Different Treatable Traits in Symptomatic Airways Disease. Respirology. 2018;23:105-.
392. Gaudino R, Murri V, Piazza M, et al. Idiopathic central precocious puberty (ICPP), adult lung function and asthma. Horm Res Paediatr. 2013;1:174.
393. Giovannini M, Valli M, Ribuffo V, et al. Relationship between Methacholine Challenge Testing and exhaled nitric oxide in adult patients with suspected bronchial asthma. Eur Ann Allergy Clin Immunol. 2014;46(3):109-13.
394. Godnic-Cvar J. [Normal reactivity and hyperreactivity in the bronchi induced by respiratory irritants]. Plucne Bolesti. 1990;42(1-2):30-2.
395. Greulich T, Sterk PJ, Hamm D, et al. An electronic nose can distinguish between different asthma phenotypes. Eur Respir J. 2013;42.
396. Guevara-Rattray E, Garden F, Reddel HK, et al. Obstructive Lung Diseases in Australian Adults: A Latent Class Analysis. American Journal of Respiratory and Critical Care Medicine. 2018;197.
397. Guly HR. Frostbite and other cold injuries in the heroic age of Antarctic exploration. Wilderness Environ Med. 2012;23(4):365-70.
398. Habib SS. Relationship of fractional exhaled nitric oxide with asthma control test scoring in adult asthmatics. Annals of Thoracic Medicine. 2011;6:171.

399. Haines A, Davies E, Higgins B, et al. Identifying the most cost-effective way of diagnosing asthma in adults using multiple tests-a cost-utility analysis from the nice asthma guideline. Value in Health. 2015;18:A361.
400. Hayashi H, Tsuburai T, Watai K, et al. Can forced oscillation technique parameters predict airway hyperresponsiveness to histamine? Eur Respir J. 2014;44.
401. Hekking PP, Wagener AH, Sousa AR, et al. Prevalence And Phenotypic Characteristics Of Severe Adult-Onset Asthma In The U-Biopred Cohort. American Journal of Respiratory and Critical Care Medicine. 2014;189.
402. Hunter ML, Hui J, Knuiman M, et al. Predictors of Diagnosed Asthma and Symptoms in 'Baby-Boomers' - the Busselton Healthy Ageing Study. Respirology. 2012;17:48-.
403. ICTRP. A Phase IV, open-label, prospective, randomised clinical trial to evaluate the usefulness of measuring nitric oxide in exhaled air in the therapeutic management of adult patients with mild asthma: CENTRAL; 2012 [cited 25/05/2019]. Available from: https://www.cochranelibrary.com/central/doi/10.1002/central/CN-01856594/full.
404. Khurana S, Larj M, Saatian B, et al. Correlation Of Bronchodilator Reversibility With Exhaled Nitric Oxide Levels And Asthma Severity. American Journal of Respiratory and Critical Care Medicine. 2011;183(1).
405. Kipourou M, Michailopoulos P, Ntinapogias E, et al. Small airways disease evaluation in patients with mild/moderate asthma and correlation with bronchial hyperresponsiveness and disease control. Eur Respir J. 2018;52.
406. Kirenga B, Muttamba W, Mugenyi L, et al. A Prospective Cohort Study of Severe Asthma and Its Determinants in an African Population: The African Severe Asthma Program. American Journal of Respiratory and Critical Care Medicine. 2018;197.
407. Kobayashi H. Relationship Between Co-Morbidities and Asthma Control. American Journal of Respiratory and Critical Care Medicine. 2018;197.
408. Koruga D, Hromis S, Baletic N, et al. Evaluation bronchial hyperresponsivness in patients with persistent allergic rhinitis. Eur Respir J. 2018;52.
409. Krishnan B, Priya S. Clinical Utility of Feno Analysis in the Diagnosis of Cough-Variant Asthma. Respirology. 2017;22:7-.
410. Kulkarni T, Krishnan S, Ghosal AG, et al. DELTAFEF25-75 a surrogate marker for mild intermittent asthma: A poor man's FeNO. Eur Respir J. 2016;48.
411. Lamon T, Brouquieres D, Escamilla R, et al. Evaluation of exhaled NO measurement in the first line exploration of chronic cough in adults. Eur Respir J. 2014;44.
412. Larj MJ, Zehr L, Bhaskar J, et al. Negative Methacholine Challenge Tests In Subjects With Physician Diagnosed Asthma: Data From Soar Asthma Registry. American Journal of Respiratory and Critical Care Medicine. 2014;189.
413. Laska I, Doyle S, Jayaram L. Exploring the clinical utility of measuring reversibility in mid expiratory flow and its relationship with FEV1 reversibility in patients with asthma. Eur Respir J. 2017;50.
414. Lau CL. Use of Feno Breath Test as a Biomarker in Management of 294 Chronic Cough or Dyspnoeic Patients in a Specialist Clinic in Hong Kong. Respirology. 2017;22:91-2.
415. Leung T, Ko F, Sy H, et al. Functional ADRB2 polymorphisms are associated with asthma endophenotypes in Chinese adults but not children. Allergy: European Journal of Allergy and Clinical Immunology. 2009;90:186.
416. Luo XC, Huang WG, Liu ZJ. [Diagnostic value of measuring resistance of airway in the bronchus diastole test for asthma]. Hunan Yi Ke Da Xue Xue Bao. 2001;26(4):381-2.
417. Malinovschi A, Gislason T, Olivieri M, et al. Bronchodilator response and previous lung function decline in relation with exhaled nitric oxide levels in asthma. Eur Respir J. 2017;50.
418. Martins C, Carneiro-Leao L, Vilela A, et al. Bronchodilation assessment by oscillometry in adult asthmatic patients. Eur Respir J. 2016;48.

419. Mgaloblishvili N, Gotua M, Rukhadze M, et al. Exhaled nitric oxide and respiratory symptoms in the diagnosis of atopic asthma. Allergy. 2009;64:179-80.
420. Mohsen SM, Ben Jemaa S, El Guiche D, et al. Is Fractional exhaled nitric oxide (FeNO) test reliable in the differentiation of chronic obstructive pulmonary disease (COPD) and ACOS (asthma-COPD overlap syndrome)? Eur Respir J. 2017;50.
421. Musa O, Magzoub A, Elsony A. Prevalence of asthma symptoms in adult university students and workers in Elobeid - West Sudan. Eur Respir J. 2011;38.
422. Obtulowicz K, Laczkowska T, Kolarzyk E, et al. Obstruction of the small airways in the spirometric diagnosis of occupational bronchial asthma. J Investig Allergol Clin Immunol. 1998;8(5):300-3.
423. Oostveen E, Leemans K, Backer WD, et al. The bronchodilator responsiveness in asthmatic patients: Comparison of forced expiration and forced oscillations. American Journal of Respiratory and Critical Care Medicine. 2012;185.
424. Oyama S, Ohtani Y, Koike F, et al. Cluster Analysis Of Cough Variant Asthma Using Fot And Feno. American Journal of Respiratory and Critical Care Medicine. 2017;195.
425. Paknejad O, Hojjati SA, Pazoki M. The association between methacholine challenge test and respiratory symptoms: A study on 146 patients. Tehran University Medical Journal. 2011;68(11):662-7.
426. Pedersen SK, Ustrup AS, Barnes CB, et al. Usefulness of mannitol challenge testing for diagnosing asthma in everyday clinical practice. Eur Respir J. 2018;52.
427. Pereira A, Martins C, Fonseca J. Use of CARAT and lung function tests to assess control of asthma and rhinitis. Revista Portuguesa de Imunoalergologia. 2013;21(2):103-15.
428. Pinto PCCVRL, Martins P, Peralta I, et al. Is there any association between spirometry and impulse oscillometry in asthmatic preschool children? Eur Respir J. 2018;52:532-.
429. Polivka BJ, Cavallazzi R, Jorayeva A, et al. Predicting Positive Bronchial Challenge Test in Older Adults with Asthma. American Journal of Respiratory and Critical Care Medicine. 2018;197.
430. Rashidian A, Sazgar S, Mejia JE, et al. Residual Volume Reversibility Predicts Reversible Airflow Obstruction In Asthma Better Than Fev1 Reversibility. American Journal of Respiratory and Critical Care Medicine. 2015;191.
431. Ross J, Baptist A. Factors associated with asthma quality of life and control among older adults. Ann Allergy Asthma Immunol. 2011;1:A5.
432. Ross MJ, Rodriguez J. FEF50/FEV1 as an indicator of asthma in the athlete. Clinical Journal of Sport Medicine. 2018;28:202.
433. Scott H. The Impact of Varying Exercise Training Intensity on Clinical Asthma Outcomes and Inflammation in Adults with Asthma Cochrane Central Register of Controlled Trials (CENTRAL): CENTRAL; 2017 [updated 2019; cited 25/05/2019]. Available from: https://www.cochranelibrary.com/central/doi/10.1002/central/CN-01886029/full.
434. Sharifi A, Nazemiyeh M. Methacholine challenge test with impulse oscillometry versus spirometry: Which is more sensitive in detecting airway hyper-responsiveness (AHR?). Eur Respir J. 2018;52.
435. Shirai T, Hirai K, Akamatsu T, et al. Usefulness of the forced oscillation technique in diagnosing asthma-COPD overlap syndrome. Eur Respir J. 2016;48.
436. Siebeneichler A, Nyilas S, Schumann D, et al. Multiple breath nitrogen washout and methacholine challenge test in patients with clinical suspicion of asthma and normal lung function. Chest. 2017;151(5):38a-a.
437. Simpson J, Shrimanker R, Thulborn S, et al. Identification of pulmonary treatable traits in a real-life setting. Eur Respir J. 2018;52.
438. Stoicescu IP, Strambu I, Basca N, et al. [The results of using a simplified questionnaire for determining the prevalence of bronchial asthma]. Pneumoftiziologia. 1998;47(2):89-94.

439. Tamura K, Endo Y, Masuda T, et al. Fewer Bronchodilator Responses with Forced Oscillation Technique Following Methacholine Challenge Test Predict Asthma Exacerbations. American Journal of Respiratory and Critical Care Medicine. 2018;197.
440. Titova O, Petrova M, Vakharlovskaya M. Study of age of asthma symptom onset as a potential predictor of disease development and progression. Allergy. 2017;72:393-.
441. Toor S, Akram S, Al Mazrouei K, et al. Is it really asthma? - Appropriate assessment and testing is important for accurate diagnosis. Respiration. 2017;94:97.
442. Van Huisstede A, Elte J, Rudolphus A, et al. The association of metabolic syndrome and asthma in morbidly obese patients. Allergy: European Journal of Allergy and Clinical Immunology. 2010;92:546.
443. Van Nederveen-Bendien SA, Heijerman HGM, Van Den Ende-Van Der Velden PJW. Specific airway resistance is more sensitive in diagnosing asthma in patients with a normal bronchial challenge test (FEV1) compared to FeNO and blood eosinophils. Allergy: European Journal of Allergy and Clinical Immunology. 2016;71:89.
444. Wardzynska A, Pawelczyk M, Rywaniak J, et al. Small airways dysfunction is associated with decreased asthma control and systemic inflammation. Allergy. 2018;73:200-.
445. Wong A, Orr D, MacKay L, et al. Audit of the impact of introducing exhaled nitric oxide (FENO) monitoring to an adult asthma clinic in a district general hospital. Eur Respir J. 2011;38.
446. Yangui F, Ayari R, Triki M, et al. The relationship between exhaled nitric oxide and body mass index in controlled an uncontrolled Tunisian asthmatics. Eur Respir J. 2016;48.
447. Dicpinigaitis PV. Chronic cough due to asthma: ACCP evidende-based clinical practice guidelines. Chest. 2006;129(1):75S-9S.
448. Ludviksdottir D, Diamant Z, Alving K, et al. Clinical aspects of using exhaled NO in asthma diagnosis and management. Clin Respir J. 2012;6(4):193-207.
449. Zitt M. Clinical applications of exhaled nitric oxide for the diagnosis and management of asthma: a consensus report. Clin Ther. 2005;27(8):1238-50.
450. Harnan SE, Essat M, Gomersall T, et al. Exhaled nitric oxide in the diagnosis of asthma in adults: a systematic review. Clin Exp Allergy. 2017;47(3):410-29.
451. Machado Carrillo F, Orea Solano M, Gomez Vera J, et al. [Respiratory function tests in aspirin-induced asthma]. Rev Alerg Mex. 2000;47(6):197-203.
452. Medina RR, Gasca Bauza MR, Lopez Duran JL, et al. Changes in the lung function of asthmatic adults in treatment with cromoglycate sodium and beclometasone. [Spanish]. Revista Alergia Mexico. 2004;51(5):173-6.
453. Park SH, Lee SY, Kang SM, et al. Prediction of brobchodilator response by using FEF25-75%in adult patient with a normal spirometry result. [Korean]. Tuberculosis and Respiratory Diseases. 2011;71(3):188-94.
454. Pirogov AB, Kolosov VP, Perel'man YM, et al. Airway inflammation patterns and clinical and functional features in patients with severe uncontrolled asthma and cold-induced airway hyperresponsiveness. [Russian]. Pulmonologiya. 2016;26(6):701-7.
455. Rodriguez Medina R, Gasca Bauza MR, Lopez Duran JL, et al. [Lung functioning changes of asthmatic adults in treatment with cromolyn sodium and beclomethasone]. Rev Alerg Mex. 2004;51(5):173-6.
456. Tsurikisawa N, Oshikata C, Tsuburai T, et al. Physiologic Airway Responses to Inhaled Histamine and Acetylcholine in Patients with Mild Asthma as Analyzed by Forced Oscillation. Arerugi. 2015;64(7):952-70.
457. Winkler J, Hagert-Winkler A, Wirtz H, et al. Impulse oscillometry in the diagnosis of the severity of obstructive pulmonary disease. [German]. Pneumologie. 2009;63(5):266-75.
