# Supplementary material for: Physiological tests of small airways function in diagnosing asthma: a systematic review
Source: BMJ Open Respir Res. 2020 Dec 21;7(1):e000770. doi: 10.1136/bmjresp-2020-000770 (PMC7754643; doi:10.1136/bmjresp-2020-000770)
Supplement: Supplementary data [file bmjresp-2020-000770supp005.pdf]

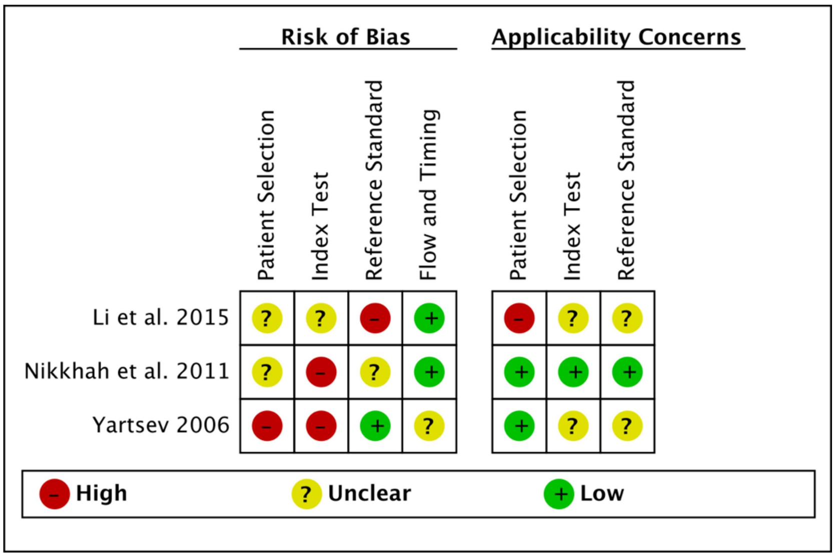

QUADAS-2 Summary

	Define the source of information	List inclusion and exclusion criteria for exposed and unexposed subjects or refer to previous publications	Indicate time period used for identifying patients	Indicate whether or not subjects were consecutive if not population–based	Indicate if evaluators of subjective components of study were masked to other aspects of the status of the participants	Describe any assessments undertaken for quality assurance purposes	Explain any patient exclusions from analysis	Describe how confounding was assessed and/or controlled	Summarize patient response rates and completeness of data collection	Clarify what follow–up, if any, was expected and the percentage of patients for which incomplete data or follow–up was obtained
Gulden et al. 2011	+	+	+	−	−	−	+	+	+	?
Koruga et al. 2017	+	+	−	−	−	−	−	−	+	−
Mendonça et al. 2011	+	−	−	−	?	−	+	?	+	?
Mori et al. 2011	+	+	+	−	−	?	+	?	+	?
Mousa & Kamal 2018	+	+	+	−	−	−	+	−	+	−
Nair et al. 2011	+	?	+	−	−	−	+	+	+	?
Son et al. 2009	+	+	+	−	−	−	+	−	+	−

AHRQ Summary
